# Anti-cancer capacity of plasma-treated PBS: effect of chemical composition on cancer cell cytotoxicity

**DOI:** 10.1038/s41598-017-16758-8

**Published:** 2017-11-28

**Authors:** Wilma Van Boxem, Jonas Van der Paal, Yury Gorbanev, Steven Vanuytsel, Evelien Smits, Sylvia Dewilde, Annemie Bogaerts

**Affiliations:** 1 Research group PLASMANT, Department of Chemistry, University of Antwerp Universiteitsplein 1, BE-2610 Wilrijk-Antwerp, Belgium; 2 Center for Oncological Research (CORE), University of Antwerp Universiteitsplein 1, BE-2610 Wilrijk-Antwerp, Belgium; 3 Research group PPES, Department of Biomedicinal Sciences, University of Antwerp Universiteitsplein 1, BE-2610 Wilrijk-Antwerp, Belgium

## Abstract

We evaluate the anti-cancer capacity of plasma-treated PBS (pPBS), by measuring the concentrations of NO_2_^−^ and H_2_O_2_ in pPBS, treated with a plasma jet, for different values of gas flow rate, gap and plasma treatment time, as well as the effect of pPBS on cancer cell cytotoxicity, for three different glioblastoma cancer cell lines, at exactly the same plasma treatment conditions. Our experiments reveal that pPBS is cytotoxic for all conditions investigated. A small variation in gap between plasma jet and liquid surface (10 mm vs 15 mm) significantly affects the chemical composition of pPBS and its anti-cancer capacity, attributed to the occurrence of discharges onto the liquid. By correlating the effect of gap, gas flow rate and plasma treatment time on the chemical composition and anti-cancer capacity of pPBS, we may conclude that H_2_O_2_ is a more important species for the anti-cancer capacity of pPBS than NO_2_^−^. We also used a 0D model, developed for plasma-liquid interactions, to elucidate the most important mechanisms for the generation of H_2_O_2_ and NO_2_^−^. Finally, we found that pPBS might be more suitable for practical applications in a clinical setting than (commonly used) plasma-activated media (PAM), because of its higher stability.

## Introduction

Cold atmospheric plasma (CAP) is gaining increasing interest for cancer treatment, but the underlying mechanisms are not yet fully understood^1–3^. In general it is believed that the reactive oxygen and nitrogen species (RONS) from the plasma are responsible for oxidative damage of biomolecules present inside the cells, eventually causing cell death^4^. These RONS are formed in significant amounts in CAPs operating directly in air, but even when the discharge gas is helium or argon, as is often the case in plasma jet devices, the plasma effluent comes in contact with the surrounding air when leaving the jet device, thus also forming RONS. Moreover, the discharge gas often contains some N_2_, O_2_ or H_2_O admixtures, thus the RONS can also directly be formed in the plasma.

The anti-cancer capacity of CAP has been reported already for many different cancer cell lines, including breast cancer^5,6^, lung cancer^7–9^, leukaemia^10^, pancreatic cancer^11^, liver cancer^12–14^, glioblastoma^15–18^, cervical cancer^19^, melanoma^18–23^, etc. Furthermore, CAP has been demonstrated to act selectively towards cancer cells, while leaving normal cells undamaged^1–4^. This selectivity has been attributed to the fact that cancer cells already have higher intracellular ROS concentrations than normal cells, and thus they have more difficulties to cope with extra oxidative damage caused by RONS from the plasma, while normal cells can defend themselves more easily, and thus reduce the oxidative stress and restore the balance^24^. In addition, other explanations have been put forward as well, such as a higher concentration of aquaporins in the plasma membrane of cancer cells, which can transport H_2_O_2_ from the plasma inside the cells^25^, and a lower concentration of cholesterol in the plasma membrane of cancer cells, which facilitates pore formation, and thus again enhances the transport of RONS from the plasma inside the cells^26,27^.

Direct CAP treatment of cancer cells or tissue also has some drawbacks, such as the need for a standardized plasma source and the way of delivery in the body, which can make it cumbersome for treatment of some organs. Therefore, plasma-activated cell media (PAM) or plasma-activated liquids (PAL) have gained increasing interest for cancer treatment^18,28–37^. Until now, the focus was mainly on the use of cell media for the plasma treatment of cancer cells. For instance, Sato *et al*. showed that PAM leads to killing of HeLa cells^28^ and Tanaka *et al*. observed that PAM selectively kills glioblastoma brain tumor cells and induces morphological changes consistent with apoptosis^15^. Vermeylen *et al*. compared CAP and PAM treatment for two melanoma and two glioblastoma cancer cell lines, in different plasma gas mixtures^18^. Recently, Canal *et al*. showed that the effect of direct treatment of cells is comparable to that of the indirect treatment of cell medium that is subsequently added to the cells^29^. Some efforts are also undertaken to exactly control the anti-cancer activity of PAM. Yan *et al*. pointed out that the killing capability of PAM can be controlled by regulating the concentration of fetal bovine serum (FBS) in media^30^. Furthermore, they showed that the addition of selected amino acids to the media can either enhance or reduce the anti-cancer effect of PAM^31,32^. Adachi *et al*. demonstrated that PAM stored at – 80 °C can remain stable for at least a week^33^. In general, PAM seems to have similar anti-cancer effects as direct CAP treatment, but it can be more generally applied, by directly injecting it into the tissue of patients.

Furthermore, instead of PAM, it could also be interesting to treat solutions with a more simple composition with plasma, and to apply these plasma-treated solutions to cancer cells. Certainly in a clinical setting, they can be seen as more standardized solutions, and they are also more suitable for the investigation of the species and mechanisms playing an important role in the anti-cancer activity of PAL, because they are not cell line dependent. Phosphate buffered saline (PBS) is an example of a simple buffered solution. Yan *et al*.^34^ showed that plasma-treated PBS (pPBS) is more stable than PAM, which is an advantage for the storage of this PAL. Only recently researchers started to use PBS for plasma treatment of cancer cells^17,32,35–38^. Wende *et al*.^35^ studied the differences between two plasma jets and the influence of ROS scavengers on the cell cytotoxicity, as well as on the concentration of H_2_O_2_. Boehm *et al*.^36^ investigated the cytotoxic and mutagenic effects of different solutions exposed to plasma, such as cell media, FBS and PBS. Yan *et al*.^32^ reported that the degradation of PAM, which they consider as the main disadvantage of PAM, can be stabilized by using pPBS. In a subsequent study, Yan *et al*.^37^ compared the anti-cancer capacity of PAM and pPBS and concluded that the vulnerability of cancer cells to PAM/pPBS is cell-dependent. Girard *et al*.^38^ studied the effect of pPBS on the viability of normal and cancer cells. Finally, Tanaka *et al*.^39^ recently used Ringer’s lactate solution as another simple solution for PAL, which was effective in killing glioblastoma cancer cells both *in vitro* and *in vivo*, due to the formation of secondary species formed via interaction of lactate with plasma RONS.

The advantages of using PAM and pPBS, or PAL in general, are rather clear, however, their anti-cancer potential is still only scarcely explored, mainly because the underlying mechanisms are largely unknown. The liquid phase chemistry of solutions exposed to plasma is quite complicated. Recently, a very comprehensive review paper was published on plasma-liquid interactions, stating the upcoming challenges, as well as the fact that there are many unresolved questions in plasma-liquid interaction^40^. Measuring the RONS concentrations in PAM and PAL is gaining increasing interest in recent years^40–44^, because they play key roles in the mechanisms taking place at cellular levels. Knowing which species are present can provide information to reveal the mechanisms taking place in the plasma treatment of cancer cells. Several RONS are suggested to play a role in the anti-cancer effect of CAP, such as OH, O_2_^−^, O, NO, H_2_O_2_, NO_2_^−^, NO_3_^−^, ONOO, NO_2_ and ONOO^−4^. However, when using PAL or PAM, only the long-lived species are of interest. H_2_O_2_^31–33,45–48^ and NO_2_^−^^38,49^ have been regarded as the key species in the anti-cancer activity of PAM. In the context of pPBS, only few studies on the effect of RONS on the cancer cells have been published. Girard *et al*.^38^ measured the concentrations of H_2_O_2_, NO_2_^−^ and NO_3_^−^ in pPBS and found that H_2_O_2_ and NO_2_^−^ have a synergistic effect on the anti-cancer capacity of pPBS, while NO_3_^−^ does not contribute to the killing of cancer cells. They also investigated the effects of treatment time and gas flow rate on these concentrations, but they only considered one or two different operating conditions for plasma treatment. Yan *et al*.^37^ showed that NO_2_^−^ alone has no killing capacity for cancer cells, while H_2_O_2_ does.

In the present paper, we focus on the effect of the long-lived species NO_2_^−^ and H_2_O_2_, produced in plasma-treated PBS (pPBS). In addition to the experiments, we also perform chemical kinetics simulations to elucidate the underlying mechanisms of NO_2_^−^ and H_2_O_2_ production and loss. We use an argon plasma jet kINPen® in this study (see Materials and Methods). It must be noted that different plasma sources have different characteristics, which may result in different nature and amount of plasma-induced RONS, and ultimately in different biological effects. As mentioned before, H_2_O_2_ and NO_2_^−^ are stated to play a key role in the anti-cancer effects of plasma treatment. Moreover, they can be easily identified, which is needed when considering many different conditions of plasma treatment. It is shown that different operating conditions (i.e. gap, treated volume, size of wells, etc.) have great influence on the concentrations of RONS and the anti-cancer capacity of PAM (e.g. ref.^31^) and therefore more efforts are needed to find the optimal operating conditions when treating liquids for plasma treatment. To identify the role of NO_2_^−^ and H_2_O_2_ in pPBS for plasma cancer treatment, we will measure their concentrations in pPBS, for several different operating conditions. Specifically, we will determine the effect of gas flow rate, gap, treatment time and occurrence of discharges on the liquid on these concentrations, and correlate the latter with the cell cytotoxicity effect of pPBS for exactly the same conditions. While the effects of (some of) these conditions have been investigated for PAM treatment in literature, to the best of our knowledge, they have never been studied for pPBS. The composition of PBS is quite different from that of PAM, and this may lead to specific trends in the effects of the operational conditions.

To correlate the chemical composition of pPBS with its anti-cancer capacity, we consider three different cell lines of glioblastoma multiforme (GBM). GBM is the most common and lethal type of primary brain tumours^50^, classified as the highest rank for tumours of the central nervous system, as issued by the WHO^51^. These tumours are characterised by a high invasiveness, molecular heterogeneity and rapid spreading throughout the brain^51^. Furthermore, they exhibit a particular resistance to surgical and medical treatment and are extremely susceptible to relapse, leading to a poor median life-expectancy of 14.6 months and a 5-year survival rate of only 9.8% when treated with conventional therapy^52^. These numbers indicate that the treatment remains palliative in most cases, demonstrating the need for alternative approaches, such as plasma treatment.

## Materials and Methods

### Plasma jet device

For the plasma treatments, we use the kINPen® IND plasma jet (INP Greifswald/neoplas tools GmbH, Greifswald, Germany). It consists of a metal cap with a pin electrode (1 mm diameter) in the middle, that is separated by a dielectric capillary (internal diameter 1.6 mm) from a grounded ring electrode^53,54^. The plasma is created by applying a sinusoidal voltage (2–6 kV_pp_) to the central electrode, with a frequency between 1.0 and 1.1 MHz, and a maximum power of 3.5 W. To limit the temperature, the device operates in burst mode, i.e., the plasma is switched on and off with a frequency of 2.5 kHz and a duty cycle of 50%. The plasma is created inside the capillary, after which the reactive plasma species are carried with the gas flow towards the open side of the device, creating a plasma effluent with length of 9–12 mm and diameter of 1 mm^53^.

### Cell culture

We evaluate the anti-cancer effect of pPBS for three human GBM cell lines (U87, U251 and LN229), which are grown in Dulbecco’s Modified Eagle Medium (DMEM) (Gibco™ DMEM, Life Technologies, 10938025), to which we add 10% fetal bovine serum (FBS) (Gibco™ FBS, Life Technologies, 10270098), 2 mM L-glutamine (Gibco™, Life Technologies, 25030081), 100 units/mL penicilline and 100 µg/mL streptomycine (Gibco™, Life Technologies, 15140163). The cells are incubated at 37 °C and 5% CO_2_.

### Plasma treatment of PBS

We apply the plasma jet to treat 2 mL PBS (pH 7.3) in a 12-well plate. We use argon gas (purity 99.999%) with a flow rate of 1–3 slm. We study the effect of three important plasma treatment parameters, i.e., gas flow rate, gap between device outlet and treated solution, and treatment time, as well as the effect of the occurrence of discharges at the liquid surface. The conditions used to link the chemical composition of pPBS with the cancer cell cytotoxicity (see below) are listed in Table 1.

**Table 1:** List of conditions. Plasma treatment conditions applied for creating pPBS, for both the chemical analysis and the effect on the cancer cell cytotoxicity.

Condition	Gas flow rate (slm)	Gap (mm)	Treatment time (min)
1	1	10	5
2	1	10	9
3	1	15	5
4	1	15	9
5	1	30	5
6	1	30	9
7	2	20	7
8	3	10	5
9	3	10	9
10	3	30	5
11	3	30	9

For conditions 1 and 2 the gap is small enough to have discharges at the liquid surface, more specifically, discharge streamers are visible between the head of the plasma jet and the liquid surface. In this case, the liquid surface acts as a third electrode, and the electrons start playing a role inside the liquid, by causing electron impact reactions. Note that when the gap is only 10 mm, but a higher flow rate of 3 slm is applied (conditions 8 and 9), no discharges take place, because the liquid is blown towards the sides of the well. For conditions 3 and 4 the gap is just large enough (i.e. 15 mm instead of 10 mm) to avoid the discharges.

Besides the conditions of Table 1, we also perform a more detailed study on the effect of plasma treatment time on the concentrations of NO_2_^−^ and H_2_O_2_ in pPBS and on the cancer cell cytotoxicity. For this purpose, we apply a gas flow rate of 1 slm, 10 mm gap (i.e., condition 1 of Table 1), and plasma treatment times of 5 min, 2 min 30 sec, 1 min 15 sec, 37.5 sec and 18.75 sec. These treatment times are obtained by so-called “diluting” the treatment time consecutively by a factor of two.

### Quantification of H_2_O_2_ in pPBS

For the detection of H_2_O_2_ we apply colourimetry, using the titanium sulphate method^55^. In acid environment, H_2_O_2_ reacts with Ti^4+^ ions, forming a yellow peroxytitanium(IV) complex (reaction R.1), which has an absorption maximum around 407 nm. This complex is stable for at least 6 hours^56^. After plasma treatment, NaN_3_ is added to this solution to avoid the destruction of H_2_O_2_ by NO_2_^−^ (reaction R.2)^57^. Indeed, NaN_3_ reacts with NO_2_^−^ according to reaction R.3, so that NO_2_^−^ disappears from the solution^58^. As these reactions occur in acidic environment, it is important to add the azide before the acid titanium(IV)-solution^56^.

R1 $${{\rm{Ti}}}^{4+}+{{\rm{H}}}_{2}{{\rm{O}}}_{2}+2{{\rm{H}}}_{2}{\rm{O}}\to {{\rm{H}}}_{2}{{\rm{TiO}}}_{4}+4{{\rm{H}}}^{+}$$

R2 $${{\rm{NO}}}_{2}^{-}+{{\rm{H}}}_{2}{{\rm{O}}}_{2}+{{\rm{H}}}^{+}\to {{\rm{NO}}}_{3}^{-}+{{\rm{H}}}_{2}{\rm{O}}+{{\rm{H}}}^{+}$$

R3 $$3{{\rm{N}}}_{3}^{-}+{{\rm{NO}}}_{2}^{-}+4{{\rm{H}}}^{+}\to 5{{\rm{N}}}_{2}+2{{\rm{H}}}_{2}{\rm{O}}$$

The analysis is performed with a ThermoFischer Genesys^TM^ 6 spectrophotometer. The cuvettes are made of quartz, and have a path length of 1 cm, a volume of 700 μL and an internal width of 2 mm. We measure the absorbance comparing with a blank solution at 400 nm. For this purpose, we prepare a solution of 80 mM NaN_3_ in PBS and a solution of 0.1 M K_2_TiO(C_2_O_4_)_2_.2H_2_O (Sigma Aldrich®, 14007) and 5 M H_2_SO_4_ in milli-Q water. For the measurements, we add 50 µL N_3_^–^solution, 200 µL pPBS and 50 µL Ti(IV)-soluton to the cuvette. Concentrations are calculated based on the extinction coefficient determined in a calibration experiment (Supplementary Figure S1).

### Quantification of NO_2_^−^ in pPBS

For measuring the NO_2_^−^ concentration, we use the Griess method^59^ (Griess Reagent Nitrite Measurement kit, Cell Signaling Technology®, 13547).

The analysis occurs in a 96-well plate with a BIO-RAD iMark^TM^ Microplate reader. 100 µL Griess reagent (1:1 sulfanilamide and *N*-(1-naftyl)-ethylenediamine) and 100 µL pPBS are added to each well. Also a blank solution is made in the well plate. The absorbance is measured in triplicate. Concentrations are calculated based on the extinction coefficient determined in a calibration experiment (Supplementary Figure S2).

### Measurement of O_3_ in pPBS

We also tried to detect O_3_ in the pPBS by means of electron paramagnetic resonance spectroscopy (EPR). The analysis occurs in Ringcaps® 50 µL capillaries with a MiniScope MS 200 (Magnettech) spectrometer. The measurements are performed by adding 4-oxo-TEMP (2,2,6,6-tetramethyl-4-piperidone) to pPBS. 4-oxo-TEMP was reported to react with ozone to produce a stable nitroxide radical 4-oxo-TEMPO (4-oxo-2,2,6,6-tetramethyl-4-piperidinyloxy).^43^ In our case, no radical formation is detected.

### Treatment of cancer cells with pPBS

Before the treatment with pPBS, the U87 and LN229 cells are plated at 3000 cells per well, while the U251 cells are plated at 1500 cells per well in 150 µL medium in a 96-well plate. These seeding densities are based on our previous experience, as they appear to be suitable for our purposes. After incubation for 24 hours at 37 °C and 5% CO_2_, the cells are treated with pPBS. For this purpose, we apply 30 µL of the pPBS to the 150 µL cells and medium present in the wells (which corresponds to a ratio of 1/6).

We also perform experiments in which catalase is added to the pPBS, as a scavenger for H_2_O_2_, in order to verify the role of H_2_O_2_ in the cancer cell cytotoxicity. For this purpose, 400 U mL^−1^ of catalase is added to the pPBS, after which the solution is stirred for 10–15 minutes. After this, 30 µL of pPBS + catalase is added to the cells in 150 µL medium. This experiment is carried out for conditions 1, 4, 6, 8, and 10.

### Treatment of cancer cells with H_2_O_2_ and/or NO_2_^−^ rich PBS

We also want to verify whether only the H_2_O_2_ or NO_2_^−^ in the pPBS is responsible for the anti-cancer capacity, or whether it is the cocktail of species that is important. For this purpose, we compare the anti-cancer capacity of pPBS with that of PBS to which H_2_O_2_ and/or NO_2_^−^ (as NaNO_2_) is added. Different solutions of PBS containing H_2_O_2_, NO_2_^−^, or a mixture of both are prepared. The concentrations of H_2_O_2_ and NO_2_^−^ are the same as in the pPBS for the conditions considered, i.e. conditions 1, 4, 6, 8, and 10. To treat the cells, 30 µL of the H_2_O_2_ and/or NO_2_^−^ rich PBS solution is added to the cells in 150 µL medium.

### Stability of pPBS

We also investigate the stability of pPBS by applying a gas flow rate of 1 slm, a gap of 15 mm and a treatment time of 5 min (i.e., condition 3 of Table 1). In a first set of experiments, we analyse the pPBS, and we add it to the cells, at fixed time steps after treatment, i.e., after 0 min, 5 min, 10 min, 30 min, 60 min and 120 min. In a second set of experiments, we assess the stability of cell medium to which we add pPBS (in a ratio of 1/6), by again analysing the chemical composition in this medium and by adding 180 μL of this medium to the cells (after removal of their original medium) at the same fixed time steps after treatment. For the chemical composition, we could only analyse the concentration of NO_2_^−^, because in the case of H_2_O_2_, the air bubbles present in the cell medium after shaking the cuvette make it impossible to measure the solution in the spectrometer.

### Cell cytotoxicity assay

After the treatment with pPBS, we analyse the cell cytotoxicity (meaning both cytostatic and cytocidal effects) by the sulforhodamine B-method (SRB)^60^. After removing the medium, the cells are fixed with 10% trichloro acetic acid (TCA). After washing away the TCA and the dead cells that are still present, we add 100 µL SRB (Sigma-Aldrich®, S1402) to each well. After thorough washing of the non-bound dye with 1% (vol/vol) acetic acid, and dissolving the bound dye with 100 µL tris-buffer (tris(hydroxymethyl)aminomethane, Sigma-Aldrich^®^, 252859), we measure the absorbance with the BIO-RAD iMark Microplate reader. The cell cytotoxicity is determined by comparing with an untreated control sample.

### Description of the model

To elucidate the underlying mechanisms responsible for the production and loss of H_2_O_2_ and NO_2_^−^ in the pPBS, we also performed computer simulations with a 0D chemical kinetics model for the plasma jet in contact with liquid water. This model is based on solving balance equations for the different species, based on production and loss terms. In total, 91 different species and 1390 different chemical reactions are included in the gas phase (plasma jet in contact with ambient air) and 35 different species and 89 different chemical reactions are considered in the liquid phase. More details about the model, and the assumptions made to mimic the experimental conditions, are given in the Supplementary Information.

### Data availability statement

All data generated or analysed during this study are included in this published article (and its Supplementary Information).

## Results and Discussion

### H_2_O_2_ and NO_2_^−^ are present at different ratios in pPBS when applying different operational conditions, but in all cases H_2_O_2_ has a higher concentration

**Figure 1:**
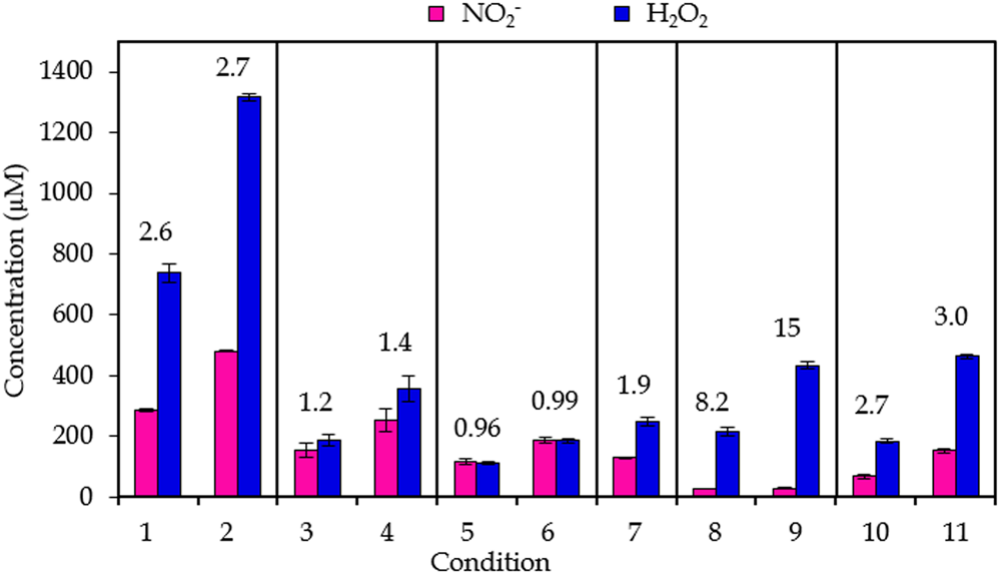
Results for chemical composition. Concentrations of NO_2_^−^ and H_2_O_2_ in pPBS at the 11 plasma treatment conditions listed in Table 1. Conditions for which only the plasma treatment time differs are indicated within one frame. The concentrations are plotted as the mean of at least three repetitions, and the error bars indicate the standard deviations of the mean. The numbers above the signals indicate the ratio of the concentration of H_2_O_2_ to the concentration of NO_2_^−^ for that condition.

Figure 1 presents the measured concentrations of NO_2_^−^ and H_2_O_2_ at the 11 conditions listed in Table 1 above. The ratios of the concentrations of NO_2_^−^ and H_2_O_2_ are also indicated in the figure. It is clear that the concentration of H_2_O_2_ is always larger than that of NO_2_^−^. Comparing conditions where the gas flow rate and gap are kept constant but only the plasma treatment time is varied, tells us that the ratio of the concentrations is kept the same, except for conditions 8 and 9, where the concentration of NO_2_^−^ is extremely low. Thus, a high gas flow rate and small gap (conditions 8 and 9) favor the formation of H_2_O_2_ compared to NO_2_^−^. Vice versa, at a low gas flow rate and a large gap (conditions 5 and 6), the concentrations of NO_2_^−^ and H_2_O_2_ are comparable, indicating that the formation of NO_2_^−^ is promoted.

The fact that different concentrations of species are formed by varying the plasma treatment conditions might be important in terms of the application, as some species will be more effective in killing the cancer cells, or might act even more selectively towards cancer cells than normal cells, in comparison to other species. Thus, by varying the plasma treatment parameters, the concentration of these particular species can be promoted.

### Chemical kinetics modelling elucidates the most important source and loss processes for the generation of H_2_O_2_ and NO_2_^−^

**Figure 2:**
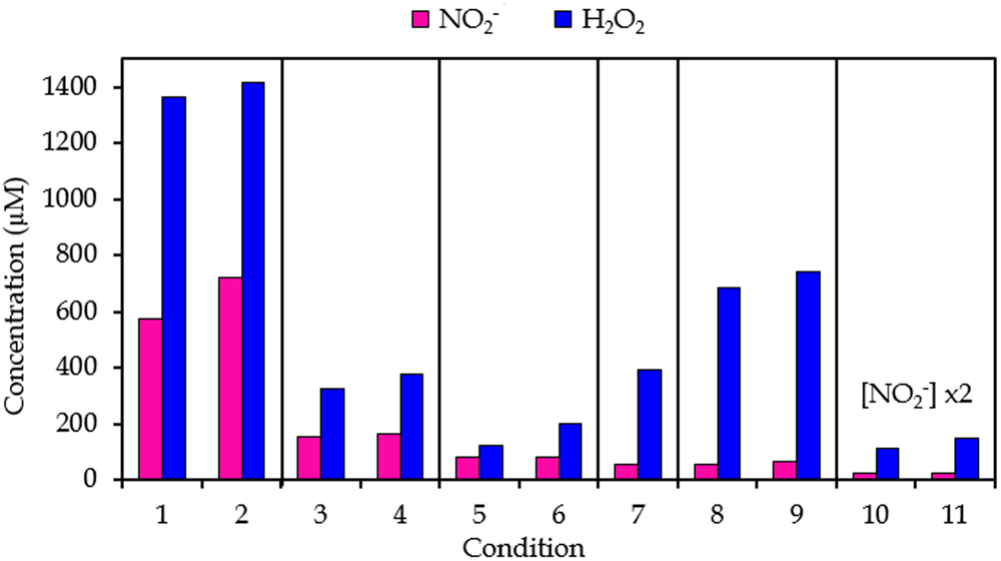
Overview of the computational results. Concentrations of NO_2_^−^ and H_2_O_2_ at the 11 plasma treatment conditions listed in Table 1, as obtained from the chemical kinetics model (see SI). Conditions for which only the plasma treatment time differs are indicated within one frame.

**Figure 3:**
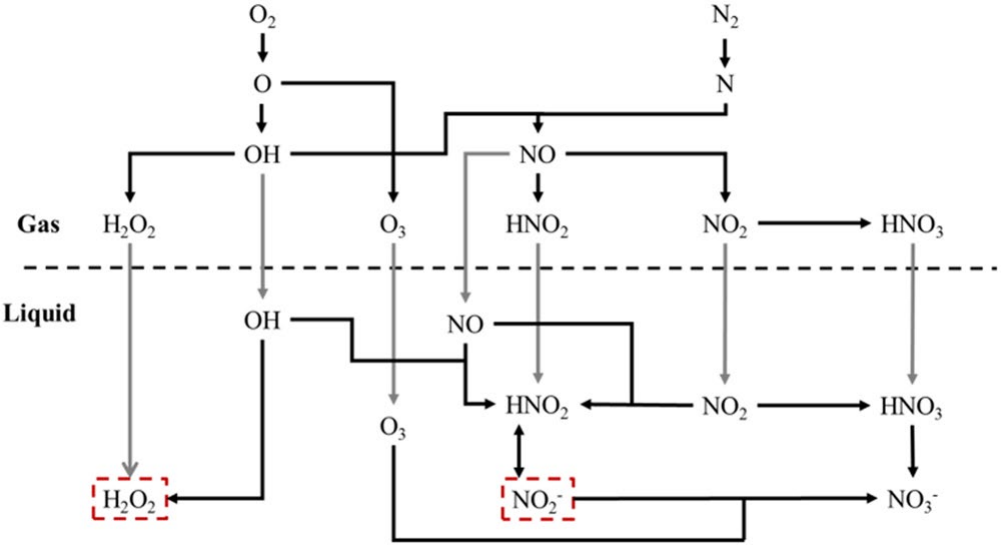
Main pathways in both the gas and liquid chemistry leading to the generation of NO_2_^−^ and H_2_O_2_. The relative contributions of the different processes (i.e. chemical reactions (black lines) or diffusion processes (gray lines)) depend on the specific treatment conditions (see text). The gas-liquid interface is illustrated by the dashed horizontal line.

Figure 2 presents the calculated liquid-phase concentrations of NO_2_^−^ and H_2_O_2_ as obtained from the model described in the Supplementary Information, at the 11 conditions listed in Table 1. When comparing these results to the experimental data of Fig. 1, a reasonable agreement is observed. Indeed, although an exact quantitative agreement cannot be expected, due to the many assumptions made when using a chemical kinetics model, similar trends are observed in both the calculations and the experiments, in terms of (i) absolute values, (ii) higher H_2_O_2_ vs NO_2_^−^ concentrations at (nearly) all conditions, and (iii) variations in concentrations as a function of plasma treatment time, gas flow rate, gap, and the presence of discharges onto the liquid surface. This indicates that the chemical kinetics model provides a realistic picture of the gas-phase and liquid-phase chemistry over the entire range of conditions investigated, and can thus be used to elucidate the underlying mechanisms at these various conditions. A general scheme which includes the main pathways leading to the generation and loss of H_2_O_2_ and NO_2_^−^, as predicted by the model, is depicted in Fig. 3.

H_2_O_2_ in the liquid phase originates back from O atoms in the plasma effluent. These radicals are mostly generated by electron-impact dissociation (R.4) or by collisions of O_2_ with excited N_2_ molecules (R.5).

R4 $${{\rm{O}}}_{2}+{{\rm{e}}}^{-}\to {\rm{O}}+{\rm{O}}+{{\rm{e}}}^{-}$$

R5 $${{\rm{O}}}_{2}+{{\rm{N}}}_{2}^{\ast }\to {\rm{O}}+{\rm{O}}+{{\rm{N}}}_{2}$$

The O atoms react further with H_2_O molecules (present in the ambient air and as impurities in the feed gas), generating OH radicals (R.6).

R6 $${\rm{O}}+{{\rm{H}}}_{2}{\rm{O}}\to {\rm{OH}}+{\rm{OH}}$$

Subsequently, two processes that lead to the generation of H_2_O_2_ in the liquid phase can occur, of which the relative contribution depends on the gap between the nozzle and the liquid. For larger gap, the OH radicals will have the time to recombine in the plasma effluent, generating H_2_O_2_ in the gas phase, which is subsequently transported into the liquid layer (R.7). For shorter gap, the gaseous OH radicals will be transport into the liquid themselves, where most of them recombine to aqueous H_2_O_2_ (R.8).

R7 $${{\rm{OH}}}_{{\rm{gas}}}+{{\rm{OH}}}_{{\rm{gas}}}+{\rm{M}}\to {{\rm{H}}}_{2}{{\rm{O}}}_{2,{\rm{gas}}}+{\rm{M}}\to {{\rm{H}}}_{2}{{\rm{O}}}_{2,{\rm{liquid}}}$$

R8 $${{\rm{OH}}}_{{\rm{gas}}}\to {{\rm{OH}}}_{{\rm{liquid}}}+{{\rm{OH}}}_{{\rm{liquid}}}\to {{\rm{H}}}_{2}{{\rm{O}}}_{2,{\rm{liquid}}}$$

The calculated relative contributions of both pathways for the different conditions are listed in Table 2. Thus, if the time needed for the plasma effluent to reach the liquid is short (i.e. short gap and high flow rate), OH radicals that are transported into the liquid layer (where they recombine) are the main source for aqueous H_2_O_2_. For longer gap and lower flow rates, aqueous H_2_O_2_ mainly originates from gaseous H_2_O_2_, which is generated by OH radical recombination in the gas phase.

**Table 2:** H_2_O_2_ generation. Contribution of H_2_O_2_ from the gas phase (R.7) and of OH radicals from the liquid phase (R.8), to the generation of aqueous H_2_O_2_.

Condition	Contribution R.7 (%)	Contribution R.8 (%)
1–2	14	86
3–4	29	71
5–6	98	2
7	15	85
8–9	2	98
10–11	41	59

The different treatment conditions not only affect the contribution of different pathways leading to the generation of H_2_O_2_, but they also affect the absolute amount of H_2_O_2_ generated in the liquid, as is clear from Figs 1 and 2. Indeed, at high flow rates and short gap, a large fraction of gaseous OH radicals seem to survive transportation into the liquid. Thus, the aqueous OH concentration will be significant, which promotes the recombination of OH radicals into H_2_O_2_ in the liquid, as this reaction rate is linearly dependent on the OH concentration squared. On the other hand, at lower flow rates and longer gaps, many of the gaseous OH radicals will already recombine in the gas phase. This recombination will, however, occur mostly with N-species (such as NO or NO_2_), because their gas density is much higher than that of O-species (~80% of ambient air consists of N_2_). This has a double effect on the aqueous H_2_O_2_ concentration: (i) the gaseous density of H_2_O_2_ will not increase upon increasing gap, so its contribution to the aqueous H_2_O_2_ is very similar in all cases (at the same flow rate), but (ii) because the aqueous OH concentration is significantly lower at larger gap, the recombination rate into H_2_O_2_ in the liquid phase will be much lower. Consequently, the aqueous H_2_O_2_ concentration will decrease upon increasing gap.

For NO_2_^−^, a similar analysis can be done. NO_2_^−^ in the liquid is in balance with HNO_2_ (see Fig. 3). The latter is mainly generated in the liquid by three processes:

R9 $${{\rm{OH}}}_{{\rm{gas}}}+{{\rm{NO}}}_{{\rm{gas}}}\to {{\rm{HNO}}}_{2,{\rm{gas}}}\to {{\rm{HNO}}}_{2,{\rm{liquid}}}$$

R10 $${{\rm{OH}}}_{{\rm{liquid}}}+{{\rm{NO}}}_{{\rm{liquid}}}\to {{\rm{HNO}}}_{2,{\rm{liquid}}}$$

R11 $${{\rm{NO}}}_{{\rm{liquid}}}+{{\rm{NO}}}_{2,{\rm{liquid}}}+{{\rm{H}}}_{2}{{\rm{O}}}_{{\rm{liquid}}}\to {{\rm{HNO}}}_{2,{\rm{liquid}}}+{{\rm{HNO}}}_{2,{\rm{liquid}}}$$

The relative contribution again depends on the treatment conditions and is shown in Table 3.

**Table 3:** NO_2_^−^ generation. Contribution of different pathways to the generation of aqueous NO_2_^−^.

Condition	Contribution R.9 (%)	Contribution R.10 (%)	Contribution R.11 (%)
1–2	67	26	7
3–4	67	25	8
5–6	68	5	27
7	63	31	6
8–9	27	68	5
10–11	53	43	4

At 1 slm flow rate, ambient air species can easily diffuse into the plasma effluent, and thus the HNO_2_ concentration will already become very large in the gas phase, which explains why it is the most important source of aqueous HNO_2_. Upon increasing flow rate, it will become more difficult for ambient air species to diffuse into the plasma effluent. Moreover, the species that are initially generated in the gas phase (i.e. OH and NO) have less time to recombine before reaching the liquid phase. Hence, the relative contribution of R.9 decreases (most prominent when compared at 10 mm gap). By increasing the gap, the species have again more time to recombine in the gas phase, and thus the contribution of R.9 will increase again compared to R.10 (most prominent when compared at 3 slm).

In order to explain the absolute concentrations of NO_2_^−^ for the different conditions investigated, we have to keep in mind that to generate any of the HNO_2_ (and thus NO_2_^−^) generating species (cf. R.9–11), both O_2_ and N_2_ are required. As mentioned before, by increasing the flow rate, the ability of these ambient air species to diffuse into the plasma effluent will drop. Therefore, the HNO_2_ concentration measured in the liquid is much more dependent on the gas flow rate than H_2_O_2_, as can be derived from Figs 1 and 2.

Moreover, Fig. 3 illustrates that the main loss process of NO_2_^−^ is the reaction with O_3_:

R12 $${{\rm{NO}}}_{2\,{\rm{liquid}}}^{-}+{{\rm{O}}}_{3}\to {{\rm{NO}}}_{3\,{\rm{liquid}}}^{-}+{{\rm{O}}}_{2}$$

By increasing the gap, the amount of O_2_ that can diffuse into the plasma effluent will rise, and thus also the amount of O_3_ generated in the plasma effluent. This gaseous O_3_ is subsequently transported into the liquid, where it will react with NO_2_^−^. This explains the drop in NO_2_^−^ concentration upon increasing gap (see Figs 1 and 2).

In summary, our model predicts that both H_2_O_2_ and NO_2_^−^ can be generated either (i) from diffusion of these species from the gas phase, or (ii) from aqueous reactions of short-lived species, and the relative contribution of both pathways strongly depends on the treatment conditions (flow rate and gap).

### H_2_O_2_, rather than NO_2_^−^, is the more important species for cancer cell cytotoxicity

**Figure 4:**
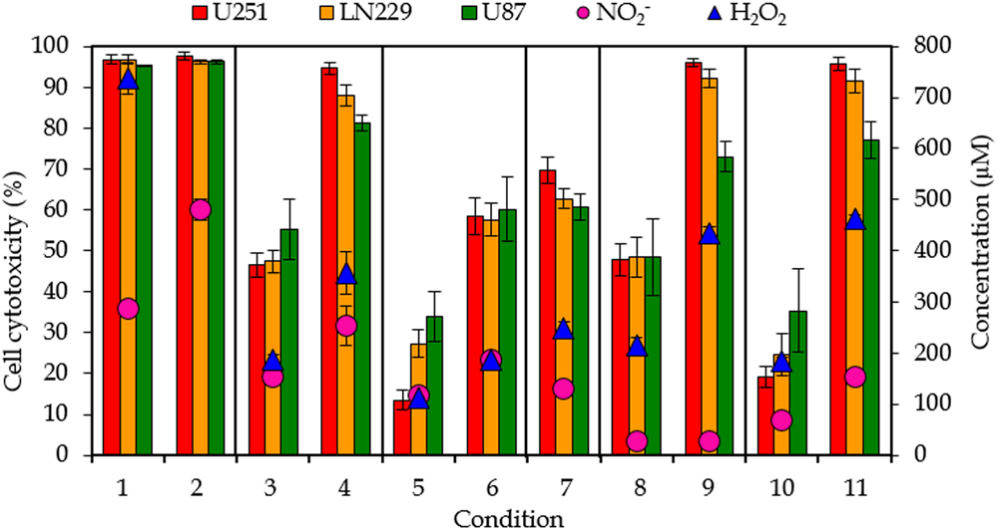
General overview of the results. Effect of pPBS on cancer cell cytotoxicity for three different GBM cell lines (U251, LN229 and U87) (left y-axis), and comparison with the concentrations of NO_2_^−^ and H_2_O_2_ in pPBS (right y-axis), for the 11 conditions listed in Table 1. Note that the H_2_O_2_ concentration in condition 2 is 1317 µM, but this is deliberately out of scale, to better evaluate the correlation between cell cytotoxicity and chemical composition for all other conditions. Conditions that only differ in plasma treatment time are indicated within one frame. The concentrations and percentages are plotted as the mean of at least three repetitions, and the error bars indicate the standard deviations of the mean.

Figure 4 presents the percentages of cell cytotoxicity for the three cell lines, along with the concentrations of NO_2_^−^ and H_2_O_2_ shown in Fig. 1, at exactly the same conditions to make the correlation between both. To evaluate whether pPBS has the same effect on cell cytotoxicity for all three cell lines, we carried out a non-parametric t-test, which indicated that for the 99% confidentiality interval only condition 11 yielded a significant difference between the cell cytotoxicity for cell lines U251 an LN229 on one hand, and for U87 on the other hand. At all other conditions, the cell cytotoxicity could be considered similar for the three cell lines. There are some differences, but they can be attributed to different air humidity during the experiments, and to a different cell growth rate for the different cell lines. Indeed, when the cell lines exhibit different growth rates after treatment, this can yield a wrong picture about their sensitivity upon treatment with pPBS, as the cell survival was evaluated by the SRB method, where the total amount of proteins is a measure for the viability with respect to an untreated control sample. However, in this study we focus on the correlation between cell cytotoxicity and NO_2_^−^ and H_2_O_2_ concentrations in pPBS for different plasma treatment conditions. Therefore, we will not further elaborate on the different sensitivity for the different cell lines.

In order to compare the concentrations of NO_2_^−^ and H_2_O_2_ with the percentage of cell cytotoxicity for all conditions investigated, it is important to note that the results of the latter are always limited to a maximum of 100% cell cytotoxicity, while the concentrations of NO_2_^−^ and H_2_O_2_ are not limited and can be higher than the concentrations needed to achieve 100% cell cytotoxicity. As a first tool for the determination of the most important species for the anti-cancer capacity of pPBS, we compare the trends between the cell cytotoxicity and the concentrations of the reactive species over all the operating conditions applied.

It is evident from Fig. 4 that the percentages of cell cytotoxicity exhibit the same trends as the concentrations of H_2_O_2_, but do not correlate with the concentrations of NO_2_^−^. Indeed, when the concentration of H_2_O_2_ raises, a higher percentage of cell cytotoxicity is obtained, and vice-versa. On the other hand, at conditions 8 and 9, there is almost no NO_2_^−^ present in pPBS, while we observe a high percentage of cell cytotoxicity. Likewise, at conditions 3 and 11, the concentration of NO_2_^−^ in pPBS is very similar, while condition 11 causes twice as much cell cytotoxicity as condition 3. Thus, we may conclude from this overview that H_2_O_2_ most probably plays a more important role in cancer cell cytotoxicity than NO_2_^−^, because the cell cytotoxicity follows the same trends as the H_2_O_2_ concentration over all the operating conditions. However, other species, like NO, NO_3_^−^ and ONOOH, can be important as well, either for killing the cancer cells, or for promoting the selectivity between cancer and normal cells (for which NO_2_^−^ may also play a role).

To further prove this statement, we perform experiments in which we add catalase to the pPBS after plasma treatment. Catalase is a scavenger for H_2_O_2_ and by adding it to the pPBS after plasma treatment, the H_2_O_2_ will be removed from the solution. For the conditions investigated (conditions 1, 4, 6, 8, and 10), adding the catalase results in no observed cell cytotoxicity in all cases (Supplementary Figure S5). This means that H_2_O_2_ indeed plays an important role for the anti-cancer activity of pPBS. From these experiments, one would expect H_2_O_2_ to be the only important species. However, further results demonstrate that this is not the case, and other plasma-induced RONS must be present in the system.

Sato *et al*.^28^ also showed that indeed H_2_O_2_ is most probably the dominant RONS inducing cancer cell death when using PAM.

### H_2_O_2_ and/or NO_2_^−^ rich PBS has not the same effect on cancer cells as pPBS

**Figure 5:**
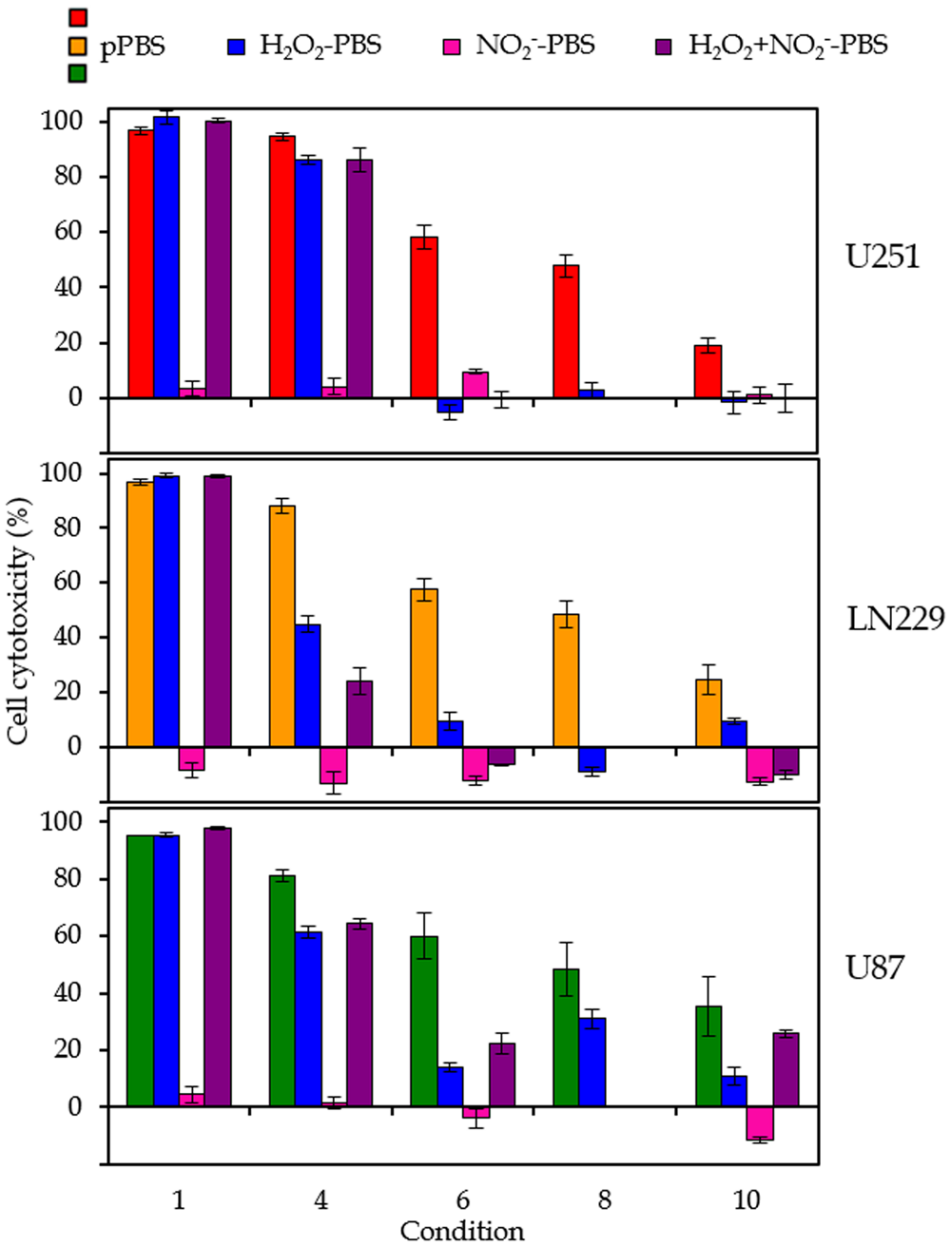
Effect of H_2_O_2_ and/or NO_2_^−^ rich PBS on cell cytotoxicity. Comparison of the effect of pPBS with H_2_O_2_ and/or NO_2_^−^ rich PBS on the cell cytotoxicity for three different GBM cell lines (U251, LN229 an U87). The concentrations of H_2_O_2_ and/or NO_2_^−^ match these in the pPBS for the conditions considered (i.e. conditions 1, 4, 6, 8 and 10, which are listed in Table 1). For condition 8, no significant amount of NO_2_^−^ is measured in the pPBS. Hence, only H_2_O_2_ rich medium is tested for that condition. The percentages are plotted as the mean of at least three repetitions, and the error bars indicate the standard deviations of the mean.

Figure 5 presents the results of the experiments where we used H_2_O_2_ and/or NO_2_^−^ rich PBS to compare the effect on cell cytotoxicity with pPBS. The concentrations of the reactive species added to PBS match these in the pPBS for the conditions considered. Firstly, it is clear that NO_2_^−^ alone has no killing effect on GBM cancer cells in any case. On the other hand, the H_2_O_2_ rich PBS is able to kill the cancer cells in all conditions for the U87 cell line. For U251 cells, the H_2_O_2_ rich PBS has only a killing effect for conditions 1 and 4, and for the LN229 cells H_2_O_2_ significantly kills cancer cells in all conditions, except 8. This reveals that H_2_O_2_ indeed contributes to the cancer cell cytotoxicity in most cases, but it cannot be the only important species. When we consider both H_2_O_2_ and NO_2_^−^ in PBS, there is mostly no additional killing effect observed, except for the U87 cell line, where in all cases a synergistic effect of H_2_O_2_ and NO_2_^−^ is observed. We can conclude that the cell lines react differently on the addition of these reactive species, while the overall effect of pPBS on the three cell lines seems comparable (see above).

This also suggests that catalase, as a scavenger of H_2_O_2_, does not only scavenge H_2_O_2_, but also other reactive species that appear to be important for the anti-cancer activity of pPBS. Indeed, catalase possibly scavenges peroxynitrite (ONOO^−^) too^61^, and possibly other RONS.

Girard *et al*.^38^ reported a synergistic effect of H_2_O_2_ and NO_2_^−^ when using pPBS on colon cancer and melanoma cells, which is in correlation with our results for the U87 cell line. Yan *et al*.^31^ demonstrated that adding only H_2_O_2_ to cancer cells does not have the same effect as PAM treatment, suggesting that other RONS also play a role. Kurake *et al*.^49^ reported that other RONS than H_2_O_2_ and NO_2_^−^ also participate in the anti-cancer capacity when using PAM, and found a synergistic effect of H_2_O_2_ and NO_2_^−^ on U251 cells. However, they used another plasma source that produces significantly higher relative amounts of NO_2_^−^ compared to the plasma jet used in our study. Indeed, while we always have higher amounts of H_2_O_2_ present in the pPBS, they measure NO_2_^−^ concentrations that are 30 times greater than that of H_2_O_2_.

Overall, it is unambiguous that H_2_O_2_ plays one of the major roles in the anti-cancer effect of pPBS, whereas NO_2_^−^ is less important. However, the cell cytotoxicity of pPBS cannot be explained by H_2_O_2_ rich PBS alone, and other reactive species must contribute to the cell cytotoxicity as well, when using pPBS.

### Do the concentrations of H_2_O_2_ and NO_2_^−^, as well as the cell cytotoxicity increase linearly with plasma treatment time?

From Figs 1 and 4 we can easily deduce the effect of the plasma treatment time on the concentrations of NO_2_^−^ and H_2_O_2_ in the liquid and on the cell cytotoxicity, because the conditions that only differ in treatment time are plotted within one frame. The ratios of the concentrations and cell cytotoxicity, for a plasma treatment time of 9 and 5 min, are listed in Table 4 for the different conditions investigated. For the results of chemical composition, it is clear that this ratio is close to 1.8 (i.e., the ratio of the treatment times) for most conditions, except for conditions 10 and 11 (for both NO_2_^−^ and H_2_O_2_) and for conditions 8 and 9 (only for NO_2_^−^). However, for these last conditions, the concentrations of NO_2_^−^ are very low, making the results less reliable. It is yet unclear why the ratio at conditions 10 and 11 (i.e., high flow rate and large gap) is different from 1.8.

**Table 4:** Effect of plasma treatment time. Ratios of the concentrations of NO_2_^−^ and H_2_O_2_ in pPBS and of the cell cytotoxicity for the three cell lines, for the plasma treatment times of 9 and 5 minutes, for the different conditions listed in Table 1. A ratio of 1.8 suggests a linear increase of the concentrations with treatment time. Results that significantly deviate from the linear increase are indicated with an asteriks. The values are given as the ratios of mean values of at least three repetitions ± the standard deviation.

Condition	Gas flow rate (slm)	Gap (mm)	NO_2_^−^	H_2_O_2_	U251	LN229	U87
1–2	1	10	1.67 ± 0.03	1.79 ± 0.08	/	/	/
3–4	1	15	1.6 ± 0.3	1.9 ± 0.3	2.0 ± 0.1	1.9 ± 0.1	1.5 ± 0.2
5–6	1	30	1.6 ± 0.2	1.6 ± 0.1	4.3 ± 0.8*	2.1 ± 0.3	1.8 ± 0.4
8–9	3	10	1.1 ± 0.1*	2.0 ± 0.1	2.0 ± 0.2	1.9 ± 0.2	1.5 ± 0.3
10–11	3	30	2.3 ± 0.2*	2.5 ± 0.1*	5.0 ± 0.7*	3.7 ± 0.8*	2.2 ± 0.6*

For the effect of the plasma treatment time on the cancer cell survival, we cannot use the results of conditions 1 and 2, because they both reach 100% cell cytotoxicity (cf. Figure 4). It is clear from Table 4 that the ratios of the percentage cell cytotoxicity are again close to 1.8, except for conditions 10 and 11 (which is in agreement with the chemical composition), and for U251 for conditions 5 and 6, which may be due to experimental variations.

**Figure 6:**
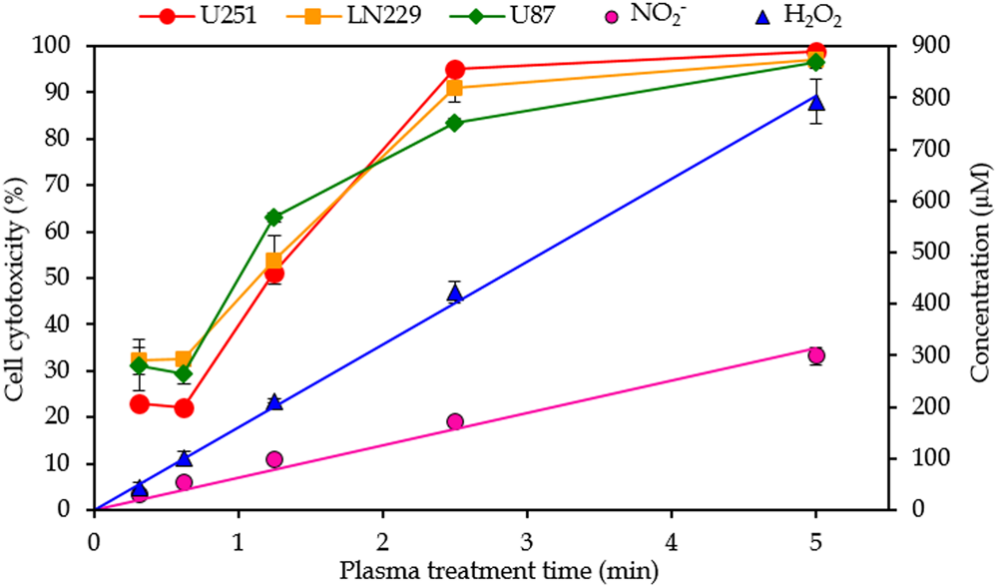
Effect of plasma treatment time. Concentrations of NO_2_^−^ and H_2_O_2_ in pPBS (right y-axis) and cell cytotoxicity (left y-axis) as a function of plasma treatment time, for a gap of 10 mm and a gas flow rate of 1 slm (i.e., condition 1 of Table 1). The concentrations and percentages are plotted as the mean of at least three repetitions, and the error bars indicate the standard deviations of the mean.

A more detailed study of the effect of plasma treatment time was performed as well, and the results are plotted in Fig. 6. The concentrations of NO_2_^−^ and H_2_O_2_ indeed rise linearly with increasing plasma treatment time, until a time of about 5 minutes, at the conditions of gap and gas flow rate investigated here (i.e., 10 mm and 1 slm). We did not apply longer treatment times here, but Table 4 indeed suggests that this linearity more or less continues up to 9 minutes. Yan *et al*.^31^ also demonstrated a linear rise in the concentrations of RNS and H_2_O_2_ in PAM for a treatment time up to 2 minutes, when using a helium plasma jet. We may conclude from our results that up to 9 minutes of plasma treatment no saturation of the plasma species in the pPBS occurs.

For the effect on the cell cytotoxicity, the shortest plasma treatment time (18.75 sec) apparently yields more or less the same cell cytotoxicity as the treatment time of 37.5 sec. Hence, it seems that small amounts of plasma species in the pPBS already give some cell cytotoxicity but that the effect is not linear here. On the other hand, a treatment time of 5 minutes results in 100% cell cytotoxicity at these conditions, so this data point cannot be considered for evaluating the linearity, as 100% cell cytotoxicity will be reached already somewhere between 2.5 and 5 min. It seems that the cell cytotoxicity does not increase linearly with treatment time for the U87 cell line, while the U251 and LN229 cell lines exhibit a more linear behaviour, although this is again based on only three data points. Several studies also reported a more or less linear effect of the plasma treatment time on cell death^19^ or RONS concentrations^14,38,49^ when using PAM, albeit also with some deviations.

### Increasing the gap results in lower concentrations of NO_2_^−^ and H_2_O_2_ and less cell cytotoxicity

**Figure 7:**
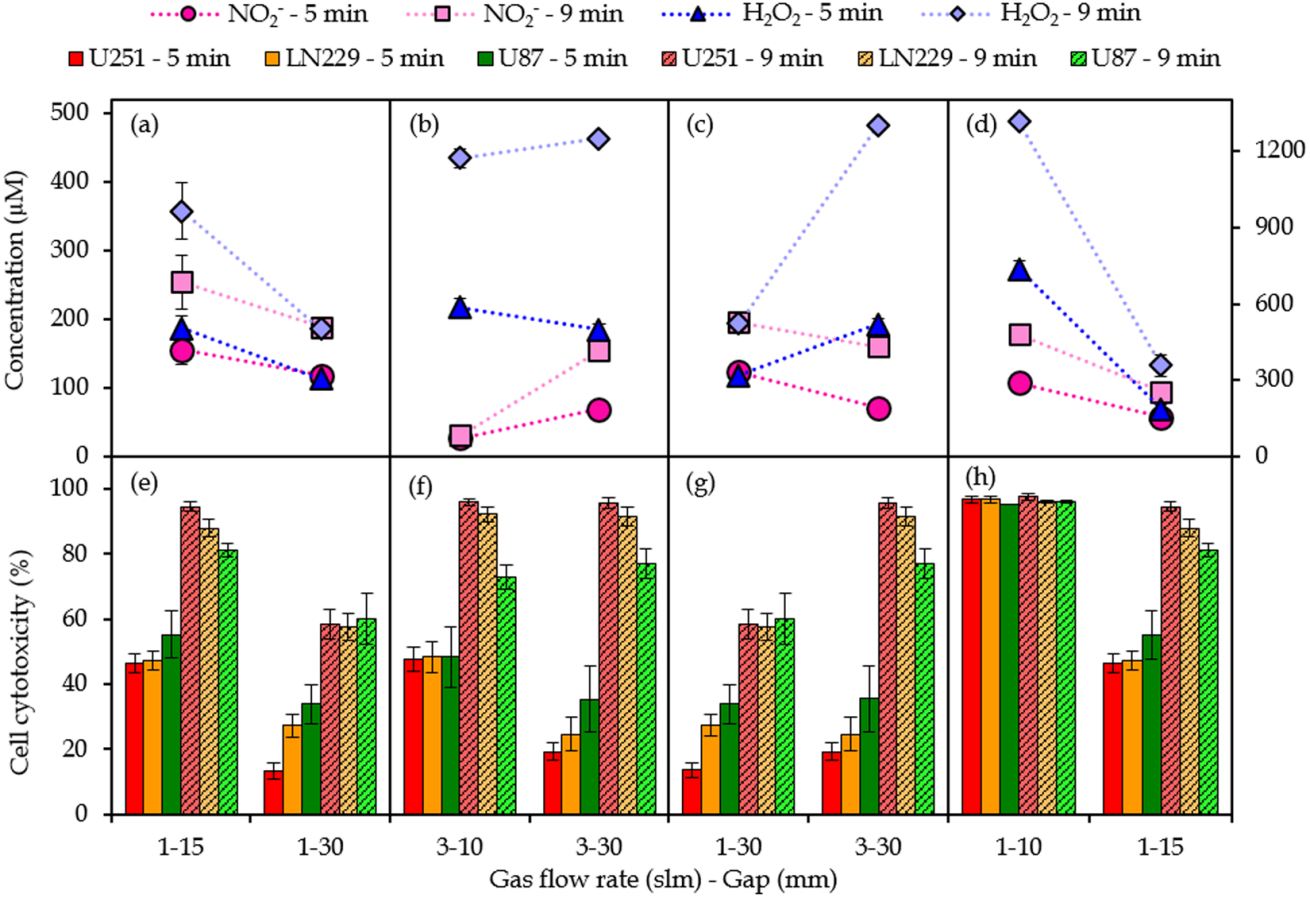
Effect of plasma treatment parameters. Effect of the gap (**a**,**b**,**e**,**f**), gas flow rate (**c**,**g**) and discharges at the liquid surface (**d**,**h**) on the concentrations of NO_2_^−^ and H_2_O_2_ in pPBS (**a**–**d**) and on the cell cytotoxicity for the three cell lines (**e**–**f**). For the effect of the discharges, the concentrations of NO_2_^−^ and H_2_O_2_ are plotted on the right y-axis. The concentrations and percentages are plotted as the mean of at least three repetitions, and the error bars indicate the standard deviations of the mean.

The effect of the gap on the chemical composition of pPBS and the cell cytotoxicity is illustrated in Fig. 7(a,b,e,f). At a gas flow rate of 1 slm (Fig. 7(a)), both the concentrations of NO_2_^−^ and H_2_O_2_ show a drop upon increasing gap from 15 to 30 mm, although the drop is more pronounced for H_2_O_2_ than for NO_2_^−^. Yan *et al*.^31^ and Takeda *et al*.^62^ also reported such a drop upon increasing gap when using PAM. This drop can be explained because the reactive plasma species are present in the gas phase for a longer time, so they have more chance to get lost upon reaction with other species. Hence, a lower amount of reactive plasma species (i.e. OH radicals for the H_2_O_2_ generation, and OH and NO radicals for the NO_2_^−^ generation, cf. modelling results above) arrive in the liquid, explaining the lower concentrations of NO_2_^−^ and H_2_O_2_. For NO_2_^−^ an additional explanation is provided by the modelling results above. A higher gap results in the formation of more O_3_, which will react with NO_2_^−^ in the liquid, lowering its concentration. On the other hand, at a gas flow rate of 3 slm (Fig. 7(b)), the NO_2_^−^ concentration rises upon increasing gap. It seems that this flow rate is high enough to transport the RONS into the liquid, without the risk for them to get lost by reactions. However, as the concentration of NO_2_^−^ at conditions 8 and 9 is extremely low, we cannot draw conclusions from this trend. For H_2_O_2_ the effect of the gap looks negligible at a gas flow rate of 3 slm. As mentioned above, we expect the concentration of H_2_O_2_ to be dependent on the reactions of OH radicals, either in the gas or in the liquid phase. At a gas flow rate of 3 slm, increasing the gap will have no significant effect on the H_2_O_2_ concentration, because the gas flow rate is high enough to enable the remaining OH radicals (not yet recombined to H_2_O_2_ in the gas phase) to reach the liquid, independent from the gap.

Figure 7(e,f) also demonstrates that a larger gap results in less cell cytotoxicity, both at 1 and 3 slm. Only for a treatment time of 9 min with a gas flow rate of 3 slm, the gap seems to have no effect. This might be correlated to the fact that the concentration of H_2_O_2_ at these conditions is also the same, thus pointing towards the important role of H_2_O_2_ in causing cell cytotoxicity. In general, the effect of the gap seems to be the same on the cell cytotoxicity and on the chemical composition, for both NO_2_^−^ and H_2_O_2_, but is more pronounced at lower flow rates than at higher flow rates.

### The gas flow rate has an opposite effect on NO_2_^−^ and H_2_O_2_, while the cell cytotoxicity acts similarly as H_2_O_2_

Figure 7(c,g) depicts the effect of the gas flow rate on the chemical composition, as well as on the cell cytotoxicity. A higher gas flow rate yields a slight drop in NO_2_^−^ concentration, but a rise in H_2_O_2_ concentration. Since the concentration of NO_2_^−^ depends on the surrounding air molecules (see above), we can state that a higher gas flow rate limits the number of air molecules that can come in contact with the plasma effluent, leading to a lower NO_2_^−^ concentration in the liquid. On the other hand, our modelling results reveal that the H_2_O_2_ formation depends on (i) the H_2_O_2_ from the gas phase reaching the liquid phase, and (ii) the recombination of two OH radicals in the liquid. As mentioned before, at high flow rates, the fraction of gaseous OH radicals that reach the liquid is large. Since the generation of H_2_O_2_ molecules is linearly dependent on the OH concentration squared, a significantly higher amount of H_2_O_2_ will be detected. It is clear that the H_2_O_2_ concentration is more affected by the gas flow rate than by the gap, while both effects are comparable for the NO_2_^−^ concentration.

A higher gas flow rate results in more cell cytotoxicity for all three cell lines. This correlates well with the trend of the H_2_O_2_ concentration, again suggesting that the cell cytotoxicity is primarily caused by H_2_O_2_ and less (or not) by NO_2_^−^.

Girard *et al*.^38^ reported lower concentrations of both H_2_O_2_ and NO_2_^−^ when increasing the gas flow rate. However, they applied only two conditions to conclude this, and the gas flow rate was 8 times higher in the second condition. The fact that we consider lower variations in gas flow rate can explain these different findings, demonstrating again the importance of the operating conditions during the plasma treatment.

### The occurrence of discharges on the liquid has a great effect on the concentrations of reactive species and on the cell cytotoxicity

Finally, the effect of discharges at the liquid surface is presented in Fig. 7(d,h). Enlarging the gap till a distance where no discharges take place anymore at the liquid surface (i.e., 15 mm instead of 10 mm) has a striking effect on the concentrations in the liquid phase. Note that the difference shown in this figure is the combination of two effects, i.e., a large gap and the disappearance of discharges. However, it was clear from Fig. 7(a,b,e,f) that enlarging the gap in the absence of discharges at the surface only has a minor effect. Therefore, we can conclude that the effect shown in Fig. 7(d,h) is predominantly due to the presence of surface discharges. The NO_2_^−^ concentration drops with a factor 2, while the H_2_O_2_ concentration even drops with a factor 4 upon disappearance of these discharges. For H_2_O_2_, the occurrence of discharges onto the liquid surface results in the formation of more OH radicals in the liquid (see Table 2), due to electron impact reactions with the water molecules in PBS. As predicted by our model, this results in higher H_2_O_2_ concentrations (see above). On the other hand, the relative contributions for the formation of NO_2_^−^ do not change with or without the presence of discharges (see Table 3). The electron impact reactions with the liquid have an overall rising effect on the reactive species present in both gas and liquid phase, resulting in higher NO_2_^−^ concentrations. As the raise of H_2_O_2_ is higher than that of NO_2_^−^, we can conclude that the generation of H_2_O_2_ depends more on the generation of reactive species out of electron impact reactions.

As expected, the occurrence of discharges at the liquid surface results in more cell cytotoxicity. Indeed, a gap of 10 mm and flow rate of 1 slm results in 100% cell cytotoxicity, while the same flow rate but a gap of 15 mm results in ca. 50% and 90% cell cytotoxicity for a plasma treatment time of 5 and 9 min, respectively. This indicates that the occurrence of discharges at the liquid surface yields at least twice as much cell cytotoxicity, which is in agreement with the results for the chemical composition of pPBS.

### PBS might be a better storage solution for RONS than cell media

**Figure 8:**
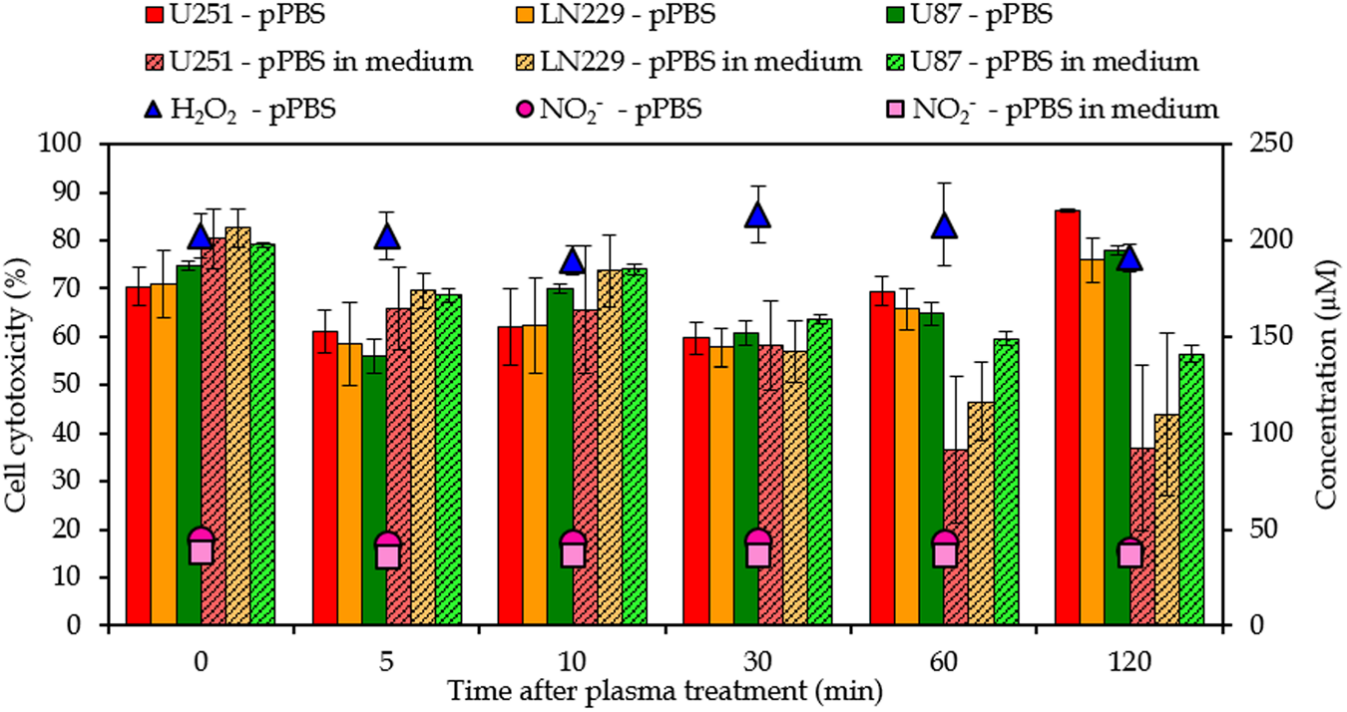
Stability of pPBS and pPBS with medium. Concentrations of NO_2_^−^ and H_2_O_2_ (right y-axis) and cell cytotoxicity of the three cell lines (left y-axis) as a function of time after treatment, to evaluate the stability of pPBS and of pPBS with medium, for a gap of 15 mm and a gas flow rate of 1 slm (i.e., condition 3 of Table 1). The concentrations and percentages are plotted as the mean of at least three repetitions, and the error bars indicate the standard deviations of the mean.

As mentioned in the Introduction, it is reported in literature that PAM can be stored during 7 days at a temperature of −80 °C^33^. Here we investigate the stability of pPBS at room temperature for a period of 2 hours. This would be convenient for practical applications of pPBS in a clinical setting. For this purpose, we measured the concentrations of NO_2_^−^ and H_2_O_2_ in pPBS at fixed times after plasma treatment (see Fig. 8). The concentrations of NO_2_^−^ and H_2_O_2_ in pPBS obviously remain constant during at least 2 hours. We also added the pPBS to the cancer cells at these times after treatment and see that the effect on the cell cytotoxicity remains constant as well (see Fig. 8).

In addition, the concentration of NO_2_^−^ was also determined when pPBS was added to the cell medium immediately after treatment, showing again a stable concentration for at least 2 hours (see Fig. 8). Unfortunately, we could not determine the concentration of H_2_O_2_ in this case, as mentioned above. However, when pPBS is added to cell medium directly after treatment, the percentage cell cytotoxicity is lower when pPBS + medium is applied to the cancer cells after 30–60 min. This suggests that the reactive species in pPBS responsible for cell death react with organic molecules in the medium, so that pPBS loses part of its anti-cancer capacity when it is added to cell medium. As the concentration of NO_2_^−^ in pPBS + medium remains constant, the reduced cell cytotoxicity will be attributed to other reactive species, possibly H_2_O_2_, as it was reported that the H_2_O_2_ concentration in PAM drops when kept at room temperature^33,63^. Indeed, Yan *et al*.^34^ investigated the instability of PAM and found that H_2_O_2_ reacts with cysteine and methionine resulting in a lower anti-cancer capacity of the cell media. As mentioned above, we could not measure the H_2_O_2_ concentraton in medium + pPBS, so we cannot conclude whether a drop in H_2_O_2_ concentraton is causing this reduced cell cytotoxicity. As the concentration of H_2_O_2_ in pPBS (without medium) remains stable at room temperature for at least 2 hours (see Fig. 8), we tentatively conclude that PBS is a better way of storage for plasma species than cell medium itself, and thus, that pPBS could be more suitable for practical applications in a clinical setting than PAM.

## Conclusion

We measured the chemical composition, more specifically the concentrations of NO_2_^−^ and H_2_O_2_, of plasma-treated PBS (pPBS) with the kINPen^®^IND plasma jet, for different values of gas flow rate, gap and plasma treatment time, and we also evaluated the effect of this pPBS on cancer cell cytotoxicity for three different GBM cell lines, i.e., U251, LN229 and U87, at exactly the same plasma treatment conditions. This should allow us to draw conclusions on the anti-cancer capacity of pPBS, and on the role of the two above-mentioned plasma species in pPBS for killing cancer cells.

First, it is clear from our experiments that varying the operating conditions during plasma treatment leads to different ratios of H_2_O_2_ and NO_2_^−^ concentrations in pPBS, which can be important to consider when we know the exact role of individual species on cancer cell cytotoxicity and on their selectivity towards normal cells. To explain the generation processes of both H_2_O_2_ and NO_2_^−^, we used a 0D chemical kinetics model. We found that the H_2_O_2_ concentration is mostly determined by the time needed for OH radicals to reach the liquid (affected by the gap and flow rate). Indeed, this determines whether the OH radicals will recombine into H_2_O_2_ (at high OH_liquid_ concentrations) or whether they will be consumed by N-species, forming HNO_2_^−^HNO_3_. For the NO_2_^−^ concentration, on the other hand, our model predicts that (i) by increasing the flow rate, fewer ambient air species are able to diffuse into the plasma plume, thereby lowering the NO_2_^−^ concentration, and (ii) by increasing the gap, more O_3_ will be generated, which will consume NO_2_^−^, hence again lowering the NO_2_^−^ concentration.

Furthermore, our experiments revealed that H_2_O_2_ is a major contributor to cancer cell cytotoxicity while NO_2_^−^ plays a minor role, but other reactive species should also play a role in the anti-cancer activity of pPBS. A synergistic effect between H_2_O_2_ and NO_2_^−^ is found for the U87 cell line, but not for the U251 and LN229 cell lines. This fact, in combination with the trends of NO_2_^−^ and H_2_O_2_ concentration and percentage cell cytotoxicity as a function of different parameters, seems to suggest that H_2_O_2_ is a more important species for the anti-cancer capacity of pPBS than NO_2_^−^, although assessing the concentrations of other plasma species, such as NO_3_^−^ and ONOO^−^, in pPBS is required in the future to draw final conclusions on the role of various plasma species in the anti-cancer capacity of pPBS. In this context, it is worth to mention that we also tried to measure the O_3_ concentration in pPBS, but that no signal could be detected. This suggests that O_3_, while it may be brought into the solution with the plasma gas, will probably rapidly disappear from it (likely back into the gas phase). Thus we may conclude that O_3_ might not play an important role in the anti-cancer capacity of pPBS.

Table 5 summarizes the results of the effects of all plasma treatment parameters on the NO_2_^−^ and H_2_O_2_ concentrations in pPBS, and on the cell cytotoxicity of the three different cancer cell lines. Increasing the gap results in lower concentrations of both NO_2_^−^ and H_2_O_2_, as well as reduced cell cytotoxicity. This is logical because the plasma species are not so efficiently transferred into the liquid, which is less effective for killing the cancer cells. Increasing the gas flow rate leads to a drop in the NO_2_^−^ concentration, because this species will not be formed so efficiently in the gas phase, as the N_2_ and O_2_ from the surrounding air come less in contact with the reactive plasma species. On the other hand, a higher gas flow rate yields a higher H_2_O_2_ concentration and also more cell cytotoxicity. This indicates that the anti-cancer capacity of pPBS is more related to the presence of H_2_O_2_ in the liquid than to the presence of NO_2_^−^. Increasing the plasma treatment time yields a more or less linear increase in both the NO_2_^−^ and H_2_O_2_ concentrations in pPBS, and in the percentage cell cytotoxicity, except for the U87 cell line, although it is a bit dangerous to draw final conclusions on the linearity for the cancer cell cytotoxicity, based on only a few data points.

**Table 5:** Summary. The effect of gap, gas flow rate, plasma treatment time, the occurrence of discharges at the liquid surface, and the stability of pPBS and pPBS with medium, on the concentrations of NO_2_^−^ and H_2_O_2_ in pPBS and on the anti-cancer capacity of pPBS for three different cancer cell lines.

	NO_2_^−^	H_2_O_2_	U251	LN229	U87
Gap ↗	↘	↘	↘	↘	↘
Gas flow rate ↗	↘	↗	↗	↗	↗
Effect of plasma treatment time: ± linear?	✓	✓	✓	✓	×
Discharges at the liquid surface	↗ × 2	↗ × 4	↗ ≥ × 2	↗ ≥ × 2	↗ ≥ × 2
Stability of pPBS?	✓	✓	✓	✓	✓
Stability of pPBS + medium?	✓	?	×	×	×

We also investigated the effect of the occurrence of discharges at the liquid surface on the NO_2_^−^ and H_2_O_2_ concentrations in pPBS and on the cancer cell cytotoxicity, and observed that these discharges have a significant influence, yielding a factor 2 and 4 higher NO_2_^−^ and H_2_O_2_ concentration in the liquid, as well as at least a factor 2 higher anti-cancer capacity of the pPBS. Indeed, these discharges allow electrons to reach the liquid and to produce more reactive species in the liquid due to electron impact reactions. This is important to realize as a small variation of the gap (in our case between 10 and 15 mm) results in either the presence or absence of discharges at the liquid surface.

Finally, the fact that pPBS is stable at room temperature for at least 2 hours, while pPBS with medium is not, indicates that pPBS might be a more suitable storage medium for practical applications in a clinical setting than PAM, which is until now most often applied.

## Electronic supplementary material

Supplementary Information
