# Supplementary material for: Anti-cancer capacity of plasma-treated PBS: effect of chemical composition on cancer cell cytotoxicity
Source: Sci Rep. 2017 Nov 28;7:16478. doi: 10.1038/s41598-017-16758-8 (PMC5705646; doi:10.1038/s41598-017-16758-8)
Supplement: Supplementary file 1 — Supplementary Information [file 41598_2017_16758_MOESM1_ESM.pdf]

Anti-cancer capacity of plasma-treated PBS: effect of chemical composition on cancer cell cytotoxicity

Wilma Van Boxem[1,*], Jonas Van der Paal[1], Yury Gorbanev[1], Steven Vanuytsel[1,2], Evelien Smits[2], Sylvia Dewilde[3] and Annemie Bogaerts[1,*]

[1] Research group PLASMANT, Department of Chemistry, University of Antwerp

Universiteitsplein 1, BE-2610 Wilrijk-Antwerp, Belgium

[2] Center for Oncological Research (CORE), University of Antwerp

Universiteitsplein 1, BE-2610 Wilrijk-Antwerp, Belgium

[3] Research group PPES, Department of Biomedicinal Sciences, University of Antwerp

Universiteitsplein 1, BE-2610 Wilrijk-Antwerp, Belgium

[*] wilma.vanboxem@uantwerpen.be

[*] annemie.bogaerts@uantwerpen.be

Supplementary Information

Calibration curves

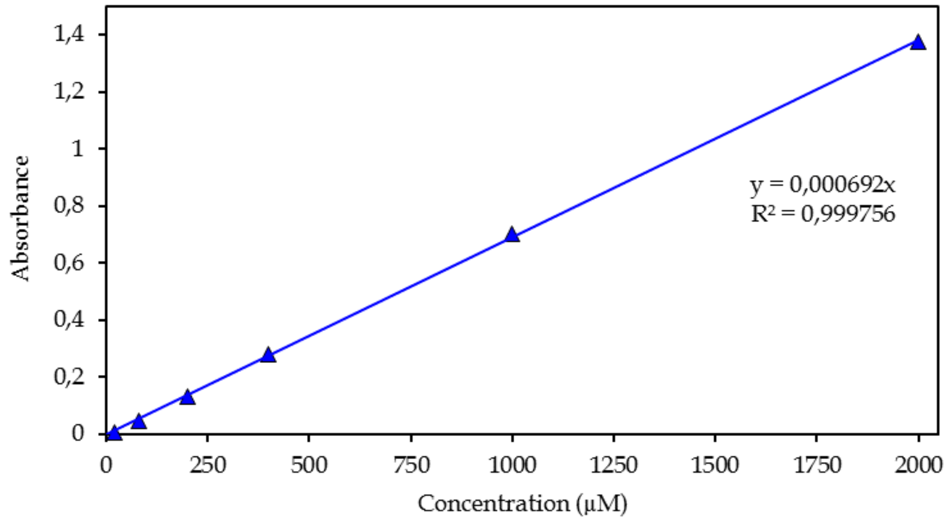

Supplementary Figure S1. Calibration curve for measurement of H_2O_2. *Standard solutions of H_2O_2 between 20 and 2000 µM were used to determine the extinction coefficient as ε = 692 L mol^{-1} cm^{-1}.*

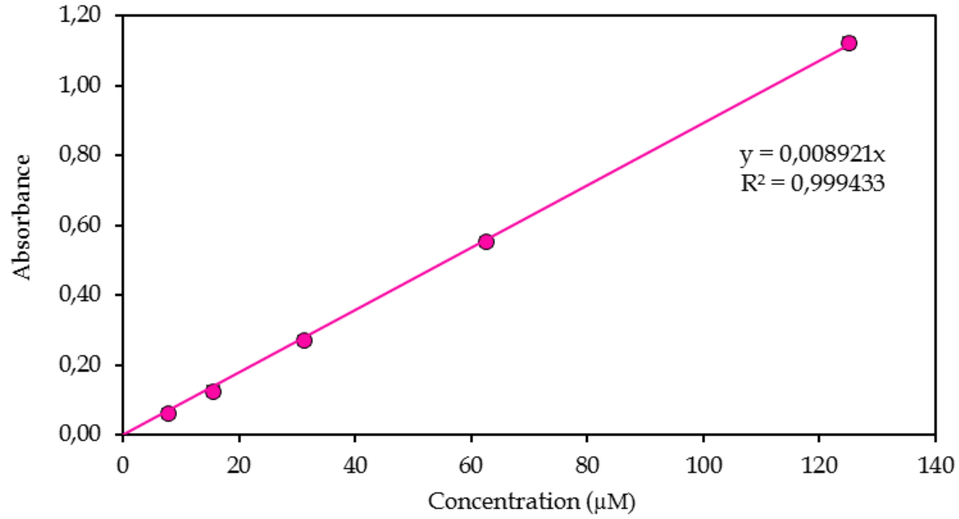

Supplementary Figure S2. Calibration curve for measurement of NO_2^-. *Standard solutions of NO_2^- between 7.8 and 125 µM were used to determine the extinction coefficient as ε = 8921 L mol^{-1} cm^{-1}.*

Model Description

Chemical kinetics model

The chemical kinetics model is based on solving a set of conservation equations (1) for all individual species included in the model (see below):

$$\frac{\partial n_s}{\partial t} = \sum_{i=1}^{j} [(a_{s,i}^R - a_{s,i}^L) R_i] \quad (1)$$

in which n_s is the density of species s (m^{-3}) , j the total number of reactions, $a_{s,i}^L$ and $a_{s,i}^R$ the stoichiometric coefficients at the left hand side and right hand side of the reaction and R_i the rate of reaction (in m^{-3} s^{-1}), given by:

$$R_i = k_i \prod_s n_s^{\alpha_{s,i}} \quad (2)$$

in which k_i is the rate coefficient (m^3 s^{-1} or m^6 s^{-1} for two-body or three-body reactions, respectively). The rate coefficients of the heavy particle reactions are either constant or dependent on the gas temperature, whereas the rate coefficients of the electron impact reactions depend on the electron temperature T_e or the reduced electric field $E/_N$ (i.e., the electric field E divided by the number density of all neutral species N, usually expressed in Td = 10^{-21} V m^2). The rate coefficients of the electron impact reactions are generally calculated according to the following equation:

$$k_i = \int_{\varepsilon_{th}}^{\infty} \sigma_i(\varepsilon) v(\varepsilon) f(\varepsilon) d\varepsilon \quad (3)$$

with ε the electron energy (usually in eV), ε_{th} the minimum threshold energy needed to induce the reaction, $v(\varepsilon)$ the velocity of the electrons (in m s^{-1}) , $\sigma_i(\varepsilon)$ the cross section of collision i (in m^2), and $f(\varepsilon)$ the (normalized) electron energy distribution function (EEDF; in eV^{-1}) calculated using a Boltzmann solver.

In this work we solve the balance equations (1) of all species by means of the ZDPlaskin code, which also has a built-in Boltzmann solver, called BOLSIG+[S.1], to calculate the EEDF and the rate coefficients of the electron impact reactions[S.2] based on a set of cross sections, the plasma composition, the gas temperature and the reduced electric field (E/N). The electric field (E; in V m^{-1}) is calculated from a given power density, using the so-called local field approximation[S.3]:

$$E = \sqrt{\frac{P}{\sigma}} \quad (4)$$

with P the input power density (in W m^{-3}) and σ the plasma conductivity (A V^{-1} m^{-1}). The plasma conductivity is estimated at the beginning of the simulations as[S.3]:

$$\sigma = \frac{e^2 n_{e,init}}{m_e v_m} \quad (5)$$

with e the elementary charge (1.6022x10^{-19} C), $n_{e,init}$ the initial electron density (in m^{-3}), m_e the electron mass (9.1094x10^{-31} kg) and v_m the collision frequency (in s^{-1}) calculated using BOLSIG+. During the simulation the plasma conductivity is calculated as[S.3]:

$$\sigma = \frac{e v_d n_e}{(\frac{E}{N})_{prev} n_0} \quad (6)$$

with v_d the electron drift velocity (in m s^{-1}), which is calculated using BOLSIG+ implemented in ZDPlaskin, and $(\frac{E}{N})_{prev}$ the reduced electric field at the previous time step (in V m^2).

Description of the plasma jet in the chemical kinetics model

In the approach of using a chemical kinetics model to simulate the kINPen plasma jet studied in this work, a cylindrical volume element is followed along the jet stream. By doing this, we assume a homogenous plasma along the radial axis (cfr. plug flow reactor). Moreover, we assume that the axial transport of mass and energy due to drift and diffusion is negligible compared to convection. Due to the very high axial flow speed (order of 10^3 cm s^{-1}) compared to the radial flow speed this seems acceptable. Upon reaching the liquid substrate, the calculated gas phase densities of all plasma species are used as input for the liquid phase module. In this module, the accumulation of species in the liquid is determined by the diffusion from gas phase species into the liquid, which is based on Henry's law, as well as by the liquid-phase chemistry. This approach, which allows us to investigate the liquid

chemistry using a chemical kinetics model, was introduced by Lietz *et al*.[S.4] The general plasma jet set-up, assumed in the model, is shown in Supplementary Figure S3.

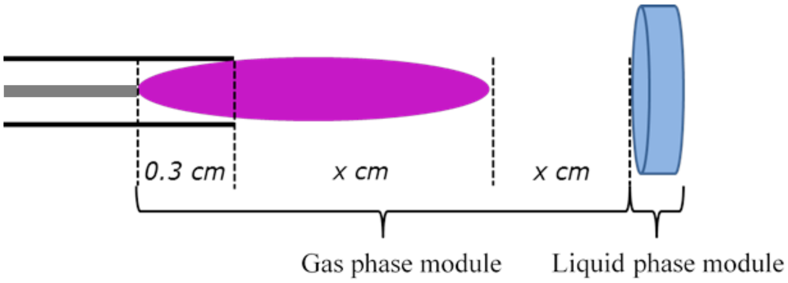

Supplementary Figure S3. Plasma jet set-up used in the chemical kinetics model. *The start of the simulation is 3 mm before the nozzle, which is at the tip of the inner electrode (thick gray line). The length of the visible plasma plume (indicated in purple) and the total distance between nozzle and liquid sample (both denoted as x cm) depend on the specific treatment conditions (see Table 1 in the main paper).*

Gas phase module

Conceptually, a chemical kinetics model calculates the density of all species as a function of time (see equation 1). However, by assuming a certain velocity profile of the feed gas, this time can be coupled to the position of the volume element along the axis, which allows us to obtain information on the species densities as a function of distance, and thus to investigate different treatment distances, as used in the experiments. An example of the gas flow velocity profile, which decreases along the axis due to gas expansion and obstruction by the relatively stationary surrounding atmosphere, is shown in Supplementary Figure S4, for a gas flow rate of 1 slm. The initial gas flow velocity, at the nozzle, is calculated based on the flow rate of the feed gas and the dimensions of the plasma jet.

Furthermore, as mentioned above, many of the gas phase reaction rate coefficients depend on the gas temperature. This means that a gas temperature profile along the plasma axis is required to calculate the exact rate of all reactions (see Supplementary Figure S4). This temperature profile is based on our experimental measurements.

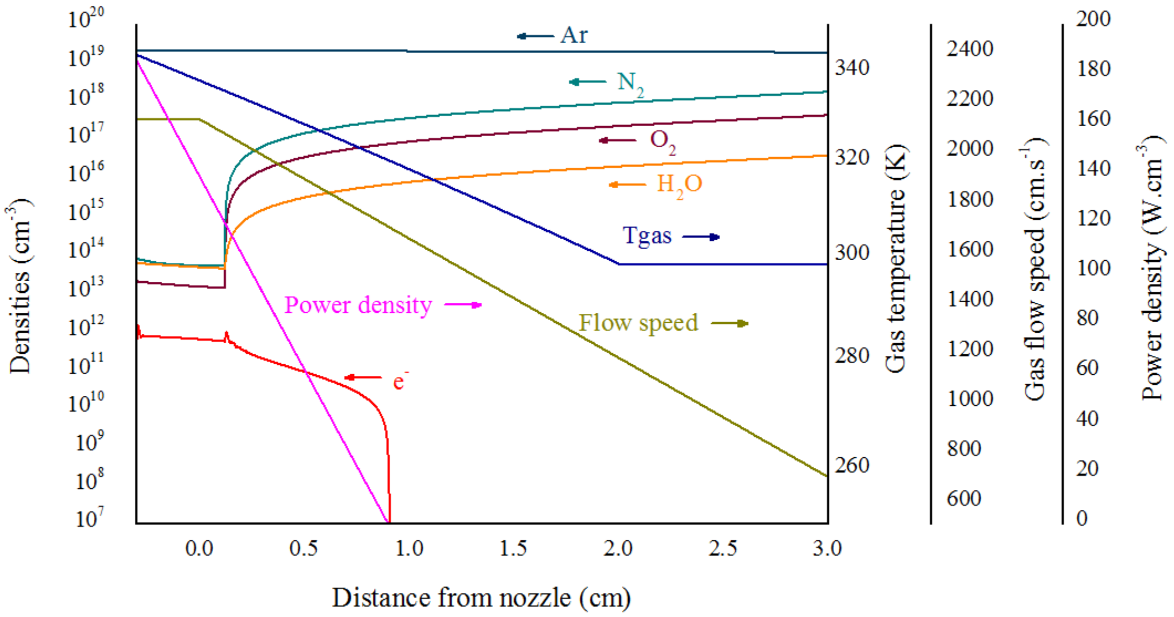

Supplementary Figure S4. *Plasma and gas characteristics as a function of the distance from the nozzle, along the plasma jet axis, for the conditions of a flow rate of 1 slm and a treatment distance of 30 mm. The profiles of the power deposition, gas temperature and humid air densities in argon due to diffusion are fitted to (i) experimental values and (ii) more detailed 2D simulations[S.5]. The electron density is calculated throughout the simulation. The grey area indicates the interior of the plasma jet, starting from the electrode tip, where the simulation starts. Note that the plasma and gas characteristics at other conditions of flow rate and treatment distance are somewhat different.*

Moreover, as the electron impact reactions depend on the EEDF, the reduced electric field is also required. As mentioned above, this reduced field is calculated based on the deposited power density, of which an example profile is also shown in Supplementary Figure S4. The maximum value of the power density is achieved at the tip of the powered electrode. Subsequently, the power density decreases linearly along the plasma axis, reaching zero at the end of the visible plasma plume, which is observed experimentally. This is chosen as the simulation results indicate that the densities of the excited species quickly drop to zero when the power density drops to zero, due to which the visible plasma plume would also be lost. The length of the plasma plume depends on the gas flow rate, based on our experimental observations, i.e., at 1 slm, the plasma plume propagates in general 9 mm into the surrounding atmosphere, whereas at 3 slm, the plasma plume has a length of 12 mm. In the case of 1 slm and a treatment distance of 10 mm, plasma discharges onto the liquid substrate were observed, as mentioned in the main paper. This means that under these conditions, a discharge between two electrodes occurs, (i) the electrode tip from the plasma jet and (ii) the liquid surface. Therefore, we assume the power density profile to rise again slightly upon reaching the liquid surface (i.e. at the end of the gas phase simulation). In all cases, the total deposited power equals 3.5 W, as is the case in the experimental treatments.

Finally, to mimic the mixing of humid air species into the effluent of the plasma jet, these species (O_2, N_2 and H_2O) are added into the effluent, assuming a certain air mixing rate (based on experimental data). The profiles of the ambient air species along the axis are also shown in Supplementary Figure S4. Note that the diffusion of ambient air species only starts after 0.12 cm in the effluent. This is because it will take some time before the ambient air species are able to diffuse up to the plasma axis. The initial densities of O_2, N_2 and H_2O inside the device (grey area in Supplementary Figure S4) originate from the impurities of the feed gas (1, 4 and 3 ppm for O_2, N_2 and H_2O, respectively), which are taken the same as the impurities present in the feed gas used in the experimental work.

The chemistry set of the gas phase reactions used in this study is largely taken from Murakami *et al*.[S.6] However, to include additional relevant biomedically active species (e.g. H_2O_2, HO_2, HNO_3 or HNO_2), we extended this chemistry set with the reactions describing the behavior of these species, adopted from the chemistry set of Van Gaens and Bogaerts[S.7], yielding a total chemistry set of 91 different species and 1390 reactions. All species included in the gas phase are shown in Supplementary Table S1.

Supplementary Table S1. *Species taken into account in the chemical kinetics model for the gas phase. The species in bold are also taken into account in the liquid phase.*

Ground state neutrals	Excited state neutrals	Charged species
Ar	$Ar(^4S[^3P_2])$, $Ar(^4S[^3P_1])$, $Ar(^4S[^3P_0])$, $Ar(^4S[^1P_1])$, Ar(4P)	**e^-**, Ar^+, Ar_2^+, ArH^+
**N, N_2**	$N(^2D)$, $N(^2P)$, $N_{2,\,vib(1\text{-}4)}$, $N_{2,rot}$, $N_2(A^3\Sigma_u^+)$, $N_2(a'^1\Sigma_u^-)$	N^+, N_2^+, N_3^+, N_4^+
**O, O_2, O_3**	$O(^1D)$, $O(^1S)$, $O_{2,vib(1\text{-}5)}$, $O_{2,rot}$, **$O_2(a^1\Delta_g)$**, $O_2(b^1\Sigma_g^+)$	O^+, O_2^+, O_4^+, O^-, **O_2^-**, O_3^-
**NO, NO_2, NO_3, N_2O, N_2O_3, N_2O_4, N_2O_5**		NO^+, NO_2^+, N_2O^+, NO^-, **NO_2^-, NO_3^-**
**H, H_2, OH, H_2O, HO_2, H_2O_2**	H^*, $H_{2,vib}$, $H_{2,rot}$, H_2^*, OH(A)	H^+, H_2^+, H_3^+, OH^+, H_2O^+, **H_3O^+**, H^-, **OH^-**, $O_2H_2O^-$, **H_2O^-, HO_2^-**
**NH, HNO, HNO_2, HNO_3**, HNO_4, **ONOOH**		$NO_2H_2O^-$, $NO_3H_2O^-$, **$ONOO^-$**

Liquid phase module

To investigate the chemistry occurring in the plasma treated liquid, a second set of species and a separate chemistry set[S.4] were included in the chemical kinetics model. First, a duplicate solvated species was added for each important gas phase species (e.g. O_{3aq} for O_3). The choice was either based on the final gas phase density of these species or their relevance for the biomedical applications. These aqueous species and the liquid reactions are restricted to the liquid module only (and are thus not taken into account in the gas phase module). The species in these two modules can only interact through the gas-liquid interphase by means of diffusion into or out of the liquid. In general, the densities of the liquid species are given by[S.4]:

$$\frac{\partial n_s}{\partial t} = \sum_{i=1}^{j}\left[\left(a_{s,i}^R - a_{s,i}^L\right)R_i\right] + \frac{D_s n_{s,g}}{\Lambda^2} f_l S_{s,l} \frac{V_p}{V_l} - max\left[0, \frac{D_s(n_{s,l} - h_s n_{s,g})}{\Lambda^2}\frac{V_p}{V_l}\right] \quad (7)$$

in which the first term is similar to the calculation of the gas phase species densities (conservation of mass; see above). The second term represents the diffusion of gas phase species into the liquid. In this term, D_s is the diffusion coefficient of gas phase species *s*, $n_{s,g}$ is the final gas phase density of species *s* and Λ is the diffusion length of the plasma. Furthermore, f_l is the fraction of the area of the plasma in contact with the liquid and $S_{s,l}$ is the sticking coefficient of species *s* on the liquid, given by:

$$S_{s,l} = \frac{h_s n_{s,g} - n_{s,l}}{h_s n_{s,g}} \quad (8)$$

in which h_s represents the Henry constant of species *s*. This sticking coefficient is only used if $n_{s,l}/n_{s,g} < h_i$ and accounts for a diminishing rate of loss of the gas phase species into the liquid as the liquid density approaches its Henry's law equilibrium values. Finally, V_p and V_l represent the volume of the plasma and the liquid, respectively. The third term of equation 7 is only non-zero if the liquid is oversaturated (i.e. if $n_{s,l}/n_{s,g} > h_i$) and represents the flux from the liquid phase into the gas phase. The Henry constants were adopted from Lietz *et al.*[S.4], whereas the diffusion coefficients were taken from Verlackt et al.[S.5]. As mentioned before, the reaction chemistry of the liquid phase is taken from Lietz *et al.*[S.4] and includes in total 35 species and 89 reactions. It is impossible to take into account the transportation of plasma species from the gas-liquid interface into the bulk of the liquid by means of this 0D chemical kinetics model, but in reality, the density of the short-lived reactive species, such as OH radicals, will drop

quickly from the interface towards the bulk. We mimic this drop in species densities in our model by decreasing the reaction rate coefficients of the reactions involving these short-lived species with longer-lived species. This approach is based on observations from more detailed 2D fluid simulations carried out in our group, in which short-lived species react mostly at the gas-liquid interface, generating more stable species, which are then rapidly transported towards the bulk of the liquid due to convection.[S.5]

Finally, it is important to mention that the liquid in our model is pure water, with 4.8 ppm O_2 and 8.9 ppm N_2 initially dissolved into it (equilibrium values with air). The experiments were performed in a buffered solution at pH 7.3, so the concentrations of H_3O^+ and OH^- in the liquid were fixed throughout the entire simulation at values which correspond to this pH.

Supplementary Table S2. *List of reactions included in the liquid module.*

Reaction	Rate coefficient
$ONOOH_{aq} + H_2O_{aq} \rightarrow H_3O^+_{aq} + ONOO^-_{aq}$	$5.0\text{x}10^{-15}$
$ONOO^-_{aq} + H_3O^+_{aq} \rightarrow H_2O_{aq} + ONOOH_{aq}$	$1.75\text{x}10^{-6}$
$HO_{2aq} + H_2O_{aq} \rightarrow H_3O^+_{aq} + O_2^-{}_{aq}$	$1.43\text{x}10^{-17}$
$H_3O^+_{aq} + O_2^-{}_{aq} \rightarrow HO_{2aq} + H_2O_{aq}$	$5.0\text{x}10^{-11}$
$HNO_{2aq} + H_2O_{aq} \rightarrow H_3O^+_{aq} + NO_2^-{}_{aq}$	$5.0\text{x}10^{-15}$
$H_3O^+_{aq} + NO_2^-{}_{aq} \rightarrow HNO_{2aq} + H_2O_{aq}$	$3.9\text{x}10^{-10}$
$HNO_{3aq} + H_2O_{aq} \rightarrow H_3O^+_{aq} + NO_3^-{}_{aq}$	$3.0\text{x}10^{-18}$
$H_3O^+_{aq} + NO_3^-{}_{aq} \rightarrow HNO_{3aq} + H_2O_{aq}$	$7.0\text{x}10^{-16}$
$HO_2NO_{2aq} + H_2O_{aq} \rightarrow O_2NO_2^-{}_{aq} + H_3O^+_{aq}$	$5.0\text{x}10^{-15}$
$O_2NO_2^-{}_{aq} + H_3O^+_{aq} \rightarrow HO_2NO_{2aq} + H_2O_{aq}$	$1.05\text{x}10^{-7}$
$OH_{aq} + H_{aq} \rightarrow H_2O_{aq}$	$3.0\text{x}10^{-11}$
$H_2O^-_{aq} + H_2O_{aq} \rightarrow H_{aq} + OH^-_{aq} + H_2O_{aq}$	$3.0\text{x}10^{-20}$
$H_2O^-_{aq} + H_{aq} \rightarrow H_{2aq} + OH^-_{aq}$	$4.0\text{x}10^{-11}$
$H_2O^-_{aq} + O_{2aq} \rightarrow H_2O_{aq} + O_2^-{}_{aq}$	$3.0\text{x}10^{-11}$
$H_2O^-_{aq} + OH_{aq} \rightarrow H_2O_{aq} + OH^-_{aq}$	$5.0\text{x}10^{-11}$
$H_2O^-_{aq} + H_2O_{2aq} \rightarrow H_2O_{aq} + OH^-_{aq} + OH_{aq}$	$2.0\text{x}10^{-13}$
$H_2O^-_{aq} + HO_2^-{}_{aq} \rightarrow OH^-_{aq} + OH^-_{aq} + OH_{aq}$	$5.0\text{x}10^{-12}$
$H_2O^-_{aq} + H_2O^-_{aq} \rightarrow H_{2aq} + OH^-_{aq} + OH^-_{aq}$	$1.0\text{x}10^{-11}$
$H_3O^+_{aq} + OH^-_{aq} \rightarrow H_2O_{aq} + H_2O_{aq}$	$5.0\text{x}10^{-15}$
$H_2O_{aq} + H_2O_{aq} \rightarrow H_3O^+_{aq} + OH^-_{aq}$	$3.02\text{x}10^{-32}$
$OH_{aq} + OH_{aq} \rightarrow H_2O_{2aq}$	$1.7\text{x}10^{-11}$
$OH_{aq} + H_{2aq} \rightarrow H_{aq} + H_2O_{aq}$	$6.0\text{x}10^{-14}$
$OH_{aq} + HO_{2aq} \rightarrow O_{2aq} + H_2O_{aq}$	$2.0\text{x}10^{-11}$
$OH_{aq} + H_2O_{2aq} \rightarrow HO_{2aq} + H_2O_{aq}$	$0.45\text{x}10^{-15}$
$OH^+_{aq}\ O_2^-{}_{aq} \rightarrow O_{2aq} + OH^-_{aq}$	$1.5\text{x}10^{-11}$
$OH_{aq} + HO_2^-{}_{aq} \rightarrow HO_{2aq} + OH^-_{aq}$	$1.5\text{x}10^{-11}$
$OH_{aq} + NO_2^-{}_{aq} \rightarrow OH^-_{aq} + NO_{2aq}$	$0.3\text{x}10^{-15}$
$OH_{aq} + NO_{aq} \rightarrow HNO_{2aq}$	$3.3\text{x}10^{-11}$
$OH_{aq} + NO_{2aq} \rightarrow HNO_{3aq}$	$2.0\text{x}10^{-11}$
$OH_{aq} + HNO_{3aq} \rightarrow NO_{3aq} + H_2O_{aq}$	$2.17\text{x}10^{-15}$
$OH_{aq} + N_2O_{aq} \rightarrow HNO_{aq} + NO_{aq}$	$3.8\text{x}10^{-17}$
$H_{aq} + H_2O_{aq} \rightarrow H_{2aq} + OH_{aq}$	$1.5\text{x}10^{-21}$
$H_{aq} + H_{aq} \rightarrow H_{2aq}$	$1.5\text{x}10^{-11}$
$H_{aq} + OH^-_{aq} \rightarrow H_2O^-_{aq}$	$3.0\text{x}10^{-14}$
$H_{aq} + HO_{2aq} \rightarrow H_2O_{2aq}$	$3.0\text{x}10^{-11}$
$H_{aq} + H_2O_{2aq} \rightarrow H_2O_{aq} + OH_{aq}$	$1.5\text{x}10^{-15}$
$H_{aq} + HNO_{aq} \rightarrow OH_{aq} + NH_{aq}$	$2.18\text{x}10^{-22}$

$H_{aq} + OH^{-}_{aq} \rightarrow E_{aq} + H_2O_{aq}$	$2.0x10^{-14}$
$H_{aq} + NO_2^{-}{}_{aq} \rightarrow NO_{aq} + OH^{-}_{aq}$	$7.5x10^{-15}$
$H_{aq} + HNO_{2aq} \rightarrow NO_{aq} + H_2O_{aq}$	$3.52x10^{-14}$
$H_{2aq} + H_2O_{2aq} \rightarrow H_{aq} + OH_{aq} + H_2O_{aq}$	$1.0x10^{-14}$
$O_{aq} + H_2O_{aq} \rightarrow OH_{aq} + OH_{aq}$	$2.2x10^{-17}$
$O_{aq} + O_{2aq} \rightarrow O_{3aq}$	$5.0x10^{-12}$
$O_{2aq} + H_{aq} \rightarrow HO_{2aq}$	$5.0x10^{-11}$
$O_2(a^1\Delta_g)_{aq} + H_2O_{aq} \rightarrow O_{2aq} + H_2O_{aq}$	$5.0x10^{-15}$
$O_2^{-}{}_{aq} + HO_{2aq} + H_2O_{aq} \rightarrow O_{2aq} + H_2O_{2aq} + OH^{-}_{aq}$	$2.68x10^{-34}$
$O_2^{-}{}_{aq} + H_2O_{2aq} \rightarrow O_{2aq} + OH_{aq} + OH^{-}_{aq}$	$2.16x10^{-24}$
$O_2^{-}{}_{aq} + NO_{aq} \rightarrow NO_3^{-}{}_{aq}$	$6.0x10^{-12}$
$O_{3aq} \rightarrow O_{2aq} + O_{aq}$	$3.0x10^{-6}$
$O_{3aq} + OH^{-}_{aq} \rightarrow O_2^{-}{}_{aq} + HO_{2aq}$	$1.16x10^{-19}$
$N_{aq} + N_{aq} \rightarrow N_{2aq}$	$5.0x10^{-14}$
$N_{aq} + H_2O_{aq} \rightarrow NH_{aq} + OH_{aq}$	$6.93x10^{-39}$
$NH_{aq} + NO_{aq} \rightarrow N_2O_{aq} + H_{aq}$	$1.3x10^{-12}$
$NH_{aq} + O_{2aq} \rightarrow HNO_{aq} + O_{aq}$	$2.3x10^{-13}$
$NO_{aq} + NO_{aq} + O_{2aq} \rightarrow NO_{2aq} + NO_{2aq}$	$6.28x10^{-36}$
$NO_{aq} + NO_{2aq} + H_2O_{aq} \rightarrow HNO_{2aq} + HNO_{2aq}$	$5.55x10^{-34}$
$NO_{aq} + HO_{2aq} \rightarrow HNO_{3aq}$	$5.33x10^{-12}$
$NO_{aq} + HO_{2aq} \rightarrow ONOOH_{aq}$	$5.33x10^{-12}$
$NO_{aq} + O_2^{-}{}_{aq} \rightarrow ONOO^{-}_{aq}$	$7.14x10^{-12}$
$2\ NO_{2aq} + 2\ H_2O_{aq} \rightarrow H_3O^{+}_{aq} + NO_3^{-}{}_{aq} + HNO_{2aq}$	$1.26x10^{-56}$
$2\ NO_{2aq} + 3\ H_2O_{aq} \rightarrow 2\ H_3O^{+}_{aq} + NO_3^{-}{}_{aq} + NO_2^{-}{}_{aq}$	$1.30x10^{-79}$
$NO_{2aq} + OH_{aq} \rightarrow ONOOH_{aq}$	$1.99x10^{-11}$
$NO_{2aq} + H_{aq} \rightarrow HNO_{2aq}$	$1.67x10^{-11}$
$NO_2^{-}{}_{aq} + O_{3aq} \rightarrow NO_3^{-}{}_{aq} + O_{2aq}$	$5.48x10^{-16}$
$NO_{3aq} + H_2O_{aq} \rightarrow HNO_{3aq} + OH_{aq}$	$4.8x10^{-14}$
$N_2O_{3aq} + H_2O_{aq} \rightarrow HNO_{2aq} + HNO_{2aq}$	$1.93x10^{-17}$
$N_2O4_{aq} + H_2O_{aq} \rightarrow HNO_{2aq} + HNO_{3aq}$	$1.33x10^{-18}$
$N_2O5^{+}_{aq}\ H_2O_{aq} \rightarrow NO_{2aq} + NO_{3aq} + H_2O_{aq}$	$1.4x10^{-19}$
$N_2O5_{aq} + H_2O_{aq} \rightarrow HNO_{3aq} + HNO_{3aq}$	$2.0x10^{-21}$
$N_2O5_{aq} + H_2O_{aq} \rightarrow ONOOH_{aq} + ONOOH_{aq}$	$2.0x10^{-21}$
$H_2O_{2aq} + NO_2^{-}{}_{aq} + H_3O^{+}_{aq} \rightarrow ONOOH_{aq} + H_2O_{aq} + H_2O_{aq}$	$3.04x10^{-39}$
$ONOOH_{aq} + H_2O_{aq} \rightarrow H_3O^{+}_{aq} + NO_3^{-}{}_{aq}$	$2.9x10^{-23}$
$ONOOH_{aq} + H_2O_{aq} \rightarrow OH_{aq} + NO_{2aq} + H_2O_{aq}$	$1.24x10^{-23}$
$HNO_{aq} + O_{2aq} \rightarrow HO_{2aq} + NO_{aq}$	$8.01x10^{-21}$
$HNO_{aq} + O_{3aq} \rightarrow O_{2aq} + HNO_{2aq}$	$9.61x10^{-15}$
$HNO_{aq} + OH_{aq} \rightarrow H_2O_{aq} + NO_{aq}$	$8.00x10^{-11}$
$O_2NO_2^{-}{}_{aq} \rightarrow NO_2^{-}{}_{aq} + O_{2aq}$	$1.0x10^{0}$
$HO_2NO_{2aq} + HNO_{2aq} \rightarrow HNO_{3aq} + HNO_{3aq}$	$1.99x10^{-20}$
$HO_2NO_{2aq} \rightarrow HNO_{2aq} + O_{2aq}$	$7.0x10^{-4}$
$HO_2NO_{2aq} \rightarrow HO_{2aq} + NO_{2aq}$	$4.6x10^{-3}$
$e_{aq} + H_2O_{aq} \rightarrow H_{aq} + OH^{-}_{aq}$	$3.04x10^{-20}$
$e_{aq} + E_{aq} + 2\ H_2O_{aq} \rightarrow H_{2aq} + 2\ OH^{-}_{aq}$	$4.096x10^{-55}$
$e_{aq} + H_{aq} + H_2O_{aq} \rightarrow H_{2aq} + OH^{-}_{aq}$	$6.4x10^{-32}$
$e_{aq} + OH_{aq} \rightarrow OH^{-}_{aq}$	$4.80x10^{-11}$
$e_{aq} + H_3O^{+}_{aq} \rightarrow H_{aq} + H_2O_{aq}$	$3.68x10^{-11}$
$e_{aq} + H_2O_{2aq} \rightarrow OH_{aq} + OH^{-}_{aq}$	$1.76x10^{-11}$
$e_{aq} + HO_2^{-}{}_{aq} + H_2O_{aq} \rightarrow OH_{aq} + 2\ OH^{-}_{aq}$	$8.96x10^{-33}$
$e_{aq} + O_{2aq} \rightarrow O_2^{-}{}_{aq}$	$3.04x10^{-11}$
$e_{aq} + H_2O_{aq} \rightarrow H_2O^{-}_{aq}$	$5.0x10^{-15}$

Catalase experiments

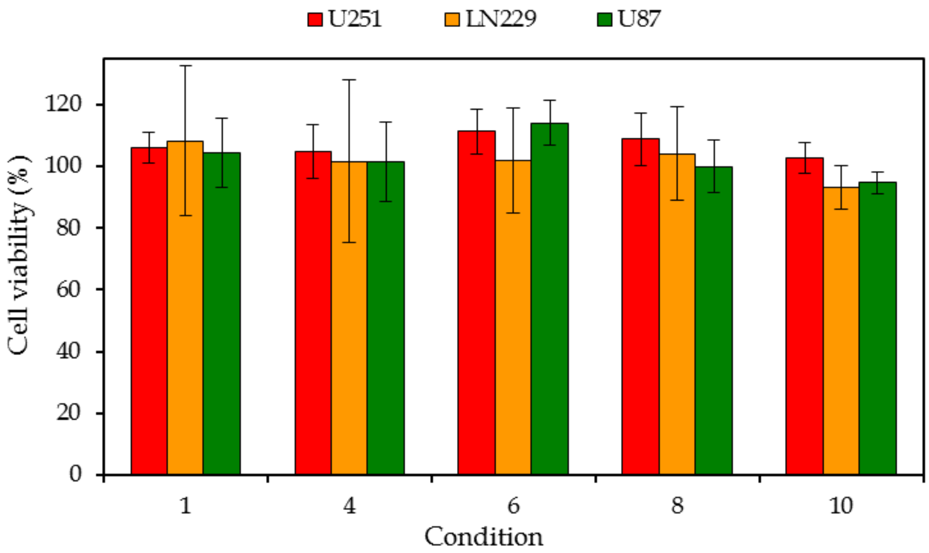

Supplementary Figure S5. Catalase experiments. *Effect of adding catalase (400 U mL^{-1}) to pPBS on the cancer cell viability for three different GBM cell lines (U251, LN229, U87). The treatment conditions are listed in Table 1. The percentages are plotted as the mean of at least three repetitions, and the error bars indicate the standard deviations of the mean. No cell cytotoxicity is observed upon addition of catalase, indicating that H_2O_2 plays an important role in the cancer cell cytotoxicity of pPBS, although it might not be the only important species, as catalase might also be able to scavenge other RONS.*

References

S.1 Hagelaar, G. J. M. & Pitchford, L.C. Solving the Boltzmann equation to obtain electron transport coefficients and rate coefficients for fluid models. *Plasma Sources Sci. Technol.* **14**, 722-733 (2005).

S.2 Pancheshnyi, S., Eismann, B., Hagelaar, G. J. M. & Pitchford, L. C. ZDPlasKin: a new tool for plasmachemical simulations. (2008).

S.3 Lieberman, M. A. & Lichtenberg, A. J. Principles of Plasma Discharges and Materials Processing (second edition) (Wiley-Interscience, 2005).

S.4 Lietz, A. M. & Kushner, M. J. Air plasma treatment of liquid covered tissue: long timescale chemistry. *J. Phys. D.: Appl. Phys.* **49**, 425204 (2016).

S.5 Verlackt, C. W. To be submitted.

S.6 Murakami, T., Niemi, K., Gans, T. & O'Connell, D. Chemical kinetics and reactive species in atmospheric pressure helium-oxygen plasmas with humid-air impurities. *Plasma Sources Sci. Technol.* **22**, 15003 (2013).

S.7 Van Gaens, W. & Bogaerts, A. Kinetic modelling for an atmospheric pressure argon plasma jet in humid air. *J. Phys. D.: Appl. Phys.* **46**, 275201 (2013).
